# Hypercholesterolemia and 27-Hydroxycholesterol Increase S100A8 and RAGE Expression in the Brain: a Link Between Cholesterol, Alarmins, and Neurodegeneration

**DOI:** 10.1007/s12035-021-02521-8

**Published:** 2021-08-27

**Authors:** Raúl Loera-Valencia, Muhammad-Al-Mustafa Ismail, Julen Goikolea, Maria Lodeiro, Laura Mateos, Ingemar Björkhem, Elena Puerta, Mariana A. Romão, Cláudio M. Gomes, Paula Merino-Serrais, Silvia Maioli, Angel Cedazo-Minguez

**Affiliations:** 1 Division of Neurogeriatrics, Department of Neurobiology Care Sciences and Society, Center for Alzheimer Research, Karolinska Institutet, Stockholm, Sweden; 2 Division of Clinical Chemistry, Department of Laboratory Medicine, Karolinska University Hospital, Huddinge, Sweden; 3 Department of Pharmacology and Toxicology, University of Navarra, Pamplona, Spain; 4 Biosystems and Integrative Sciences Institute, Faculdade de Ciências, Universidade de Lisboa, 1749-016 Lisboa, Portugal; 5 Departamento de Química E Bioquímica, Faculdade de Ciências, Universidade de Lisboa, 1749-016 Lisboa, Portugal; 6 Laboratorio Cajal de Circuitos Corticales (CTB), Universidad Politécnica de Madrid, Madrid, Spain; 7 Departamento de Neurobiología Funcional y de Sistemas, Instituto Cajal, CSIC, Madrid, Spain

**Keywords:** Alarmins, Astrocytes, Retinoid receptors, Oxysterols, Alzheimer’s disease, Sterile inflammation

## Abstract

Alterations in cholesterol metabolism in the brain have a major role in the physiology of Alzheimer’s disease (AD). Oxysterols are cholesterol metabolites with multiple implications in memory functions and in neurodegeneration. Previous studies have shown detrimental effects of cholesterol metabolites in neurons, but its effect in glial cells is unknown. We used a high-fat/high-cholesterol diet in mice to study the effects of hypercholesterolemia over the alarmin S100A8 cascade in the hippocampus. Using CYP27Tg, a transgenic mouse model, we show that the hypercholesterolemia influence on the brain is mediated by the excess of 27-hydroxycholesterol (27-OH), a cholesterol metabolite. We also employed an acute model of 27-OH intraventricular injection in the brain to study RAGE and S100A8 response. We used primary cultures of neurons and astrocytes to study the effect of high levels of 27-OH over the S100A8 alarmin cascade. We report that a high-fat/high-cholesterol diet leads to an increase in S100A8 production in the brain. In CYP27Tg, we report an increase of S100A8 and its receptor RAGE in the hippocampus under elevated 27-OH in the brain. Using siRNA, we found that 27-OH upregulation of RAGE in astrocytes and neurons is mediated by the nuclear receptor RXRγ. Silencing RXRγ in neurons prevented 27-OH-mediated upregulation of RAGE. These results show that S100A8 alarmin and RAGE respond to high levels of 27-OH in the brain in both neurons and astrocytes through RXRγ. Our study supports the notion that 27-OH mediates detrimental effects of hypercholesterolemia to the brain via alarmin signaling.

**Supplementary Information:**

The online version contains supplementary material available at 10.1007/s12035-021-02521-8.

## Background

Alzheimer’s disease (AD) is strongly associated with elevated circulating cholesterol during mid-life [1–5]. GWAS have identified risk genes involved in cholesterol metabolism in the brain including apolipoprotein E, allele epsilon 4 (APOEε4), clusterin (ApoJ), and the ATP binding cassette subfamily A member 7 (ABCA7) [6–9]. Nevertheless, cholesterol itself does not cross the blood–brain barrier (BBB), posing the question of how plasma hypercholesterolemia is linked to the risk of developing AD. Moreover, cholesterol-lowering therapies do not have a clear effect improving cognition of AD patients [10–12]. Unlike cholesterol, its side-chain oxidized metabolite, 27-hydroxycholesterol (27-OH), is able to traverse the BBB and correlates directly to circulating cholesterol levels in plasma in humans [13].

We have reported alterations in cognition observed in mice on a high-fat diet (HFD) [14] as well as in the chronically high 27-OH transgenic mice, CYP27A1 overexpressor (CYP27Tg) [15], suggesting that 27-OH may be mediated some of the deleterious effects of high peripheric cholesterol in the brain [16]. In support of this, we have shown that 27-OH affects the levels of the activity-regulated cytoskeleton-associated protein (Arc), a protein involved in long-term consolidation of memory [16].

27-OH have a proinflammatory role in atherosclerosis, being the most abundant oxysterol in atherosclerotic plaques [17]. Whether 27-OH mediates inflammation in the brain is yet not known; however, AD patients exhibit higher levels of this oxysterol in their brains and cerebrospinal fluid (CSF) [18], and inflammation is considered an important feature of AD [19].

Sterile inflammation describes a response not triggered by microbial agents but by endogenous molecules called alarmins that are released during tissue damage [20]. A number of alarmins are increased in the AD brain, including S100 calcium-binding proteins [21, 22]. We have recently reported that there is a positive feedback between Aβ and S100A8 productions in brain cells and that mice overexpressing the precursor of Aβ accumulate S100A8 aggregates in the brain prior to the appearance of Aβ plaques [23]. Furthermore, S100A8 is one of the ligands of the receptor for advanced glycation end product (RAGE) [24], expressed in almost all brain cells including microglia, neurons, and astrocytes [25, 26]. Activation of RAGE is involved in inflammatory responses, promoting synaptic dysfunction and neurodegeneration [27].

Here, we investigate the effects of a high-cholesterol diet and 27-OH on S100A8 and RAGE in the brain. Utilizing several in vivo models, primary cultures of glial cells, and neurons treated with 27-OH, we report that 27-OH increases S100A8 and RAGE levels in the brain. Most interestingly, 27-OH mediates these effects involving the retinoid X receptor gamma (RXRγ) receptor in astrocytes and neurons, while activation of the nuclear factor kappa-light-chain-enhancer of activated B cells (NFk-B) is also shown.

## Methods

### Animals

Five-to-six-week-old (C57BL/6) mice were obtained from B&K (Sollentuna, Sweden). The mice were grouped based on diet into two groups, normal chow diet (ND) or a high-fat diet (HFD) containing 21% fat and 0.15% cholesterol (R638, Lactamine, Sweden) for 9 months. Generation and breeding of the CYP27Tg mice have been described previously [28]. These mice were kept with food and water ad libitum for 12 months before sacrifice. Wild-type C57BL/6 mice were purchased from Charles River (Germany). These mice were stereotaxically injected with 1 μl of 27-OH into the lateral ventricle of both hemispheres; more details can be found below.

All mice were kept under controlled conditions of humidity and temperature on a 12-h light–dark cycle. Food and water were provided *ad libitum*. Animals were sacrificed by decapitation, and the brains immediately frozen on dry ice and stored at − 80 °C Experimental procedures involving animals were conducted in accordance with the European regulation and approved by the Swedish Board of Agriculture (ethical permits ID S33-13, extension 57–15 and 4884/2019). All efforts were made to minimize suffering or distress to experimental animals.

### Stereotaxic Injections

C57BL/6 mice were anesthetized with an isoflurane/oxygen mixture while being fixated on a stereotaxic frame (David Kopf Instruments) and placed on a heated pad at 37 °C to maintain normal body temperature. The lateral ventricle was injected bilaterally with 1 μl of solution per injection site, using a Hamilton syringe (10 μl gauge). The coordinates were set according to the Paxinos Mouse brain atlas and were 2.0 mm from the skull surface, − 0.9 mm anteroposterior, and ± 1.4 mm laterally from the bregma. The injection solution is composed of high-density lipoprotein (HDL) associated to 27-OH diluted in artificial CSF (aCSF) (RD Systems, 3525) and used at a final concentration of 10 μM. HDL diluted in aCSF was used for the control group. Incubation of 27-OH at a concentration ratio of 1:3 with HDL (Abcam, ab77881) at 37 °C for 1 h was done to produce the HDL-associated 27-OH.

### Cell Cultures and Treatments

Cerebellar tissue from 18-day-old Sprague–Dawley rat embryos was dissected. Cerebellar tissue was dissociated and seeded in Dulbecco’s Modified Eagle Medium (DMEM/F12) (Life Technologies, CA, USA) supplemented with 10% inactivated fetal bovine serum (FBS) (Life Technologies, CA, USA) in T75 plastic culture flasks (Corning, NY, USA). Cultures were incubated at 37 °C, 95% air/5% CO_2_, and culture media were replaced biweekly. Inactivated astrocyte-dominated cultures at 10–14 days were used as previously identified by immunocytochemical characterization [29]. 27-OH was obtained from Steraloids (Newport, Rhode Island, USA). Treatments were done after a 24 h period of being cultured in serum-free media and treated with 27-OH at a concentration of 1 μM for 24 h. Preincubation with 22(S)-hydroxycholesterol (22(S)-OH, 10 μM), a liver-X-receptor (LXR) inhibitor, was 3 h. Murine S100A8 was expressed in *E. coli* BL21(DE3) and purified to homogeneity using previously established protocols [30, 31] and quantitated using reported extinction coefficients [32]. Ethical consent for experiments with primary cultures was received from the regional ethical committee of Karolinska Institutet and by the Swedish Board of Agriculture (ethical permits ID S33-13, extension 57–15 and 4884/2019).

Human SH-SY5Y (ATCC® CRL-2266™) neuroblastoma cells were obtained from the American Type Culture Collection (ATCC) and cultured in a 1:1 mixture of DMEM/F-12 media and supplemented with 10% fetal bovine serum in T75 plastic culture flasks (Corning, NY, USA) at 37 ºC, with 5% CO2. For treatments, cells were seeded in 6-well plates and when confluent treated with 27-OH at 10 µM for 24 h. The nuclear and cytoplasmic extraction reagent kit NE-PER (Pierce, Rockford, IL, USA) was used to isolate the nuclear and cytosolic fractions of SH-SY5Y cells following the manufacturer’s protocol. Protease inhibitor cocktail (1:500, Sigma-Aldrich) was added freshly prepared. To quantify, immunoblotting against the nuclear marker Lamin-A/C was performed (Sigma-Aldrich SAB4200236, 1:1000).

### Small Interference RNA Transfection

#### Astrocytes

RXRγ knockdown was performed using siRNA designed by Dharmacon (ON-TARGET plus SMARTpool; L-083061–02-0020). Rat primary astrocytes (80% confluence) were transfected with a final concentration of 25 nM per well and 4 µl of DharmaFECT 3 reagent (Dharmacon) according to the manufacturer’s instructions. The efficiency of the knockdown was evaluated using mRNA levels of RXRγ.

#### Neurons

The same siRNA reagent was used for RXRγ knockdown in neurons (ON-TARGET plus SMARTpool; L-083061–02-0020). Rat primary neurons (80% confluence) at 5DIV were transfected with 25 nM per well and 4 µl of DharmaFECT 3 reagent (Dharmacon) according to the manufacturer’s instructions. After 72 h, the media was replaced for media containing the different treatments of vehicle and 27-OH 1 µM, which remained 24 h before collection. The efficiency of the knockdown was evaluated using mRNA levels of RXRγ.

### RNA Extraction and Real-Time RT-PCR

RNA extraction and real-time PCR were performed as previously described [33]. Briefly, total RNA was extracted using the RNeasy lipid tissue mini kit from Qiagen (Palo Alto, CA, USA) following the manufacturer’s instructions. Real-time PCR amplification assay for target genes was performed with a total volume of 20 μl in each well containing 10 μl of PCR Master Mix (Life Technologies, CA, USA), 2 μl of cDNA corresponding to 10 ng of RNA, and 1 μl of each TaqMan Gene Expression Assays. Relative quantification of the target genes was done using the comparative cycle threshold method, 2^−ΔΔCt^, where ΔΔCt = (*Ct*_target gene_ – *Ct*_GAPDH_)_treated_ – (*Ct*_target gene_ – *Ct*_GAPDH_)_untreated_. After the 2^−ΔΔCt^ calculations of cDNA for every sample in triplicates, expression was portrayed as a *mean* ± *SEM.*

### Immunochemistry

Immunohistochemistry was performed on coronal sections of fresh frozen brains from the hippocampus of the wild-type ND and HFD mice. The sections were fixed in cold 4% paraformaldehyde (PFA) in saline phosphate buffer (PBS, 0.1 M, pH 7.4) for 15 min and subsequently washed three times with PBS. After fixation, single immunochemistry was performed. All slices were blocked for 1 h in PBS with 0.25% Triton-X and 3% BSA. The sections were incubated overnight with the primary antibody goat anti-S100A8 (1:100; sc-48352. Santa Cruz) and then for 2 h at room temperature with the secondary antibody Alexa Fluor 594 donkey anti-goat. 4,6-Diamidino-2-phenylindole (DAPI) (Sigma, St. Louis, MO, USA) was used to identify the nuclei of cell bodies. Finally, the sections were rinsed in PBS and mounted using the fluorescence mounting medium ProLong Gold antifade reagent (Invitrogen Corporation, Carlsbad, CA, USA). The sections were thoroughly washed in PBS throughout the various stages. The primary antibody was omitted with regard to the negative control.

Immunocytochemistry was performed on glial cells from primary cultures. The cells were seeded at 50% confluence onto cover slips. Following 24 h in serum-free media, the treatments were done, and then cells were pre-fixed with 2% PFA for 2 min and fixed with 4% PFA for 20 min. Afterwards, cells were washed three times with PBS. All cover slips were blocked for 30 min in PBS with 0.1% Triton-X and 1% BSA. The primary antibodies used were goat anti-S100A8 (1:100; sc-48352, Santa Cruz) and rabbit anti-RAGE (1:100; ab3611, Abcam). The secondary antibodies used were Alexa Fluor 594 donkey anti-goat and Alexa Fluor 488 chicken anti-rabbit, respectively. Cover slips were first incubated overnight with the primary antibodies and then for 30 min at room temperature with secondary antibodies and DAPI to identify the nuclei of cell bodies. Finally rinsed in PBS, the cover slips were mounted as described above. Between the different steps, the cover slips were thoroughly washed in PBS. Again, omission of the primary antibody indicates the negative control.

Confocal imaging was performed with a Zeiss LSM 510 META confocal laser scanning system. The fluorescence of DAPI, Alexa 594, and Alexa 488 was recorded through separate channels with either Plan Apo 40 × dry (NA, 0.95) or 63 × oil (NA, 1.3) lenses.

### Cresyl Violet Staining

Fast cresyl violet staining was done to visualize the granules found in the *corpora amylacea* in the same sections used for immunohistochemistry. In brief, after the image processing, the sections were stained for 10 min with cresyl violet solution. Following staining, the sections were dehydrated and cleared with xylene. Images were taken with an optical microscope (Nikon eclipse E800M) at 20 × (NA, 0.75) under bright field optics.

### Western Blot and Densitometry Analysis

Immunoblotting was carried out in mice hippocampal tissues and primary rat culture in addition to the neuroblastoma cell line. Tissue homogenization and immunoblotting were performed as previously described [34]. The following primary antibodies were used: goat anti-S100A8 (sc-48352, Santa Cruz), rabbit anti-RAGE (ab3611, Abcam), rabbit anti-RXRγ (ab15518, Abcam), rabbit anti-NFk-B (PA1-186, Thermo Fischer), rabbit anti-actin (A2103, Sigma), and mouse anti-Lamin AC (SAB4200236, Sigma). Primary antibody incubation was followed by the incubation with the respective secondary immunoglobulin G (IgG) at 1:3000–5000 dilutions (Amersham Biosciences). Immunoreactivity was detected using the ECL detection system (Amersham Biosciences). The relative density of the immunoreactive bands was calculated from the optical density multiplied by the area of the selected band using the ImageJ 1.383 software (NIH, MA).

### Statistical Analysis

Data values are expressed as mean ± standard error mean (*SEM*). One-way ANOVA was used to compare differences between mean levels of variables among different groups, followed by Tukey’s post hoc test to compare means of every group between each other or Dunnett’s multiple comparisons to compare to the mean of a control group. Multiplicity adjusted *P* values are shown for multiple comparisons. Otherwise, unpaired *t* test or Mann–Whitney test was used. A *P* value of less than 0.05 was considered statistically significant.

## Results

### High-Fat/High-Cholesterol Diet Enhances the Expression of S100A8 in the Brain

**Fig. 1:**
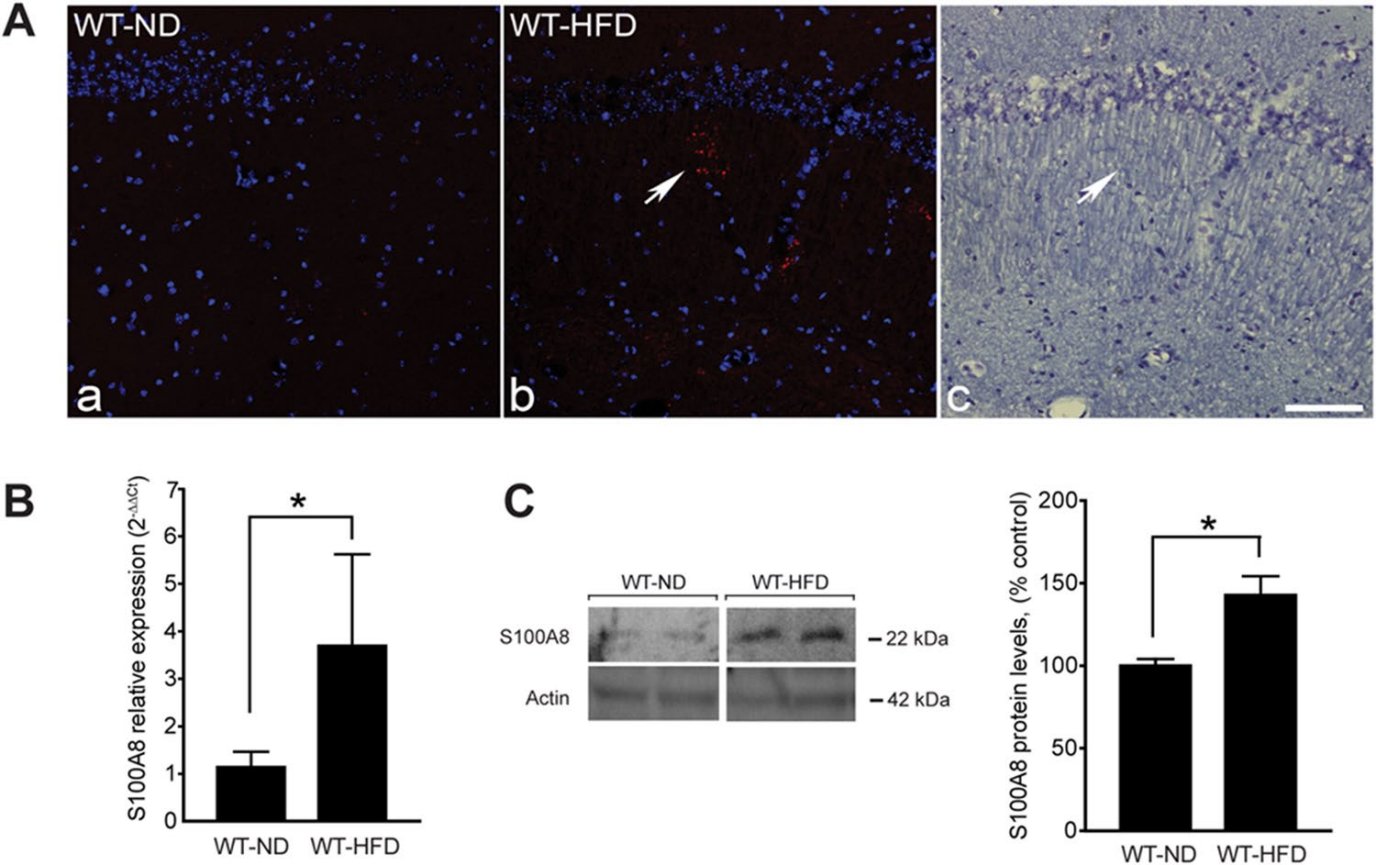
High-fat/high-cholesterol diet (HFD) and 27-OH enhance the expression of S100A8 in vivo*. A* Confocal microscopy of sections double stained with anti-S100A8 (red) and DAPI (blue) from the same field in the CA1 hippocampal region of WT mice on a normal diet (WT-ND) (**a**) or a high-fat diet (WT-HFD) (**b**). In *c*, the same section as *b* stained with cresyl violet showing, with an arrow, the S100A8 aggregates. Scale bar in **c**, 120 µm. **B** S100A8 mRNA expression levels in the hippocampus of WT-ND (*mean* = 1.34, *SEM* = 0.37, *n* = 8 animals) and WT-HFD (*mean* = 4.038, *SEM* = 1.22, *n* = 7 animals, *P* = 0.04). **C** A representative western blot showing protein levels of S100A8 of ND mice (*mean* = 100.0, *SEM* = 4.077, *n* = 4 animals) and HFD mice (*mean* = 142.8, *SEM* = 11.42, *n* = 4 animals, *P* = 0.012)

S100A8 expression was analyzed in the hippocampus of wild-type mice (WT) fed with HFD (WT-HFD) compared to a normal diet (WT-ND) through immunohistochemical analysis. Figure 1A shows an increase in S100A8 immunoreactivity in the brain of WT-HFD compared to WT-ND (insets a and b). This increase appears as extracellular granular aggregates in the *stratum oriens* and *stratum radiatum* of the hippocampus (Fig. 1A, inset b), areas of importance for learning and memory processes.

Members of the S100 protein family tend to aggregate and be part of the *corpora amylacea *(CA), which is found during the course of normal aging in the human brain [35]. Consistent with the accumulation of S100A8 seen in AD mouse models [23], cresyl violet staining revealed no overlap between the CA and the S100A8 aggregates (Fig. 1A, insets b vs. c).

Real-time PCR analysis of hippocampal samples from WT-HFD also showed an increase in S100A8 expression compared to WT-ND (Fig. 1B). By western blot, we confirmed that HFD induced increase of S100A8 in the hippocampus of mice (Fig. 1C).

### 27-OH Increases S100A8 and RAGE Expression In Vivo

**Fig. 2:**
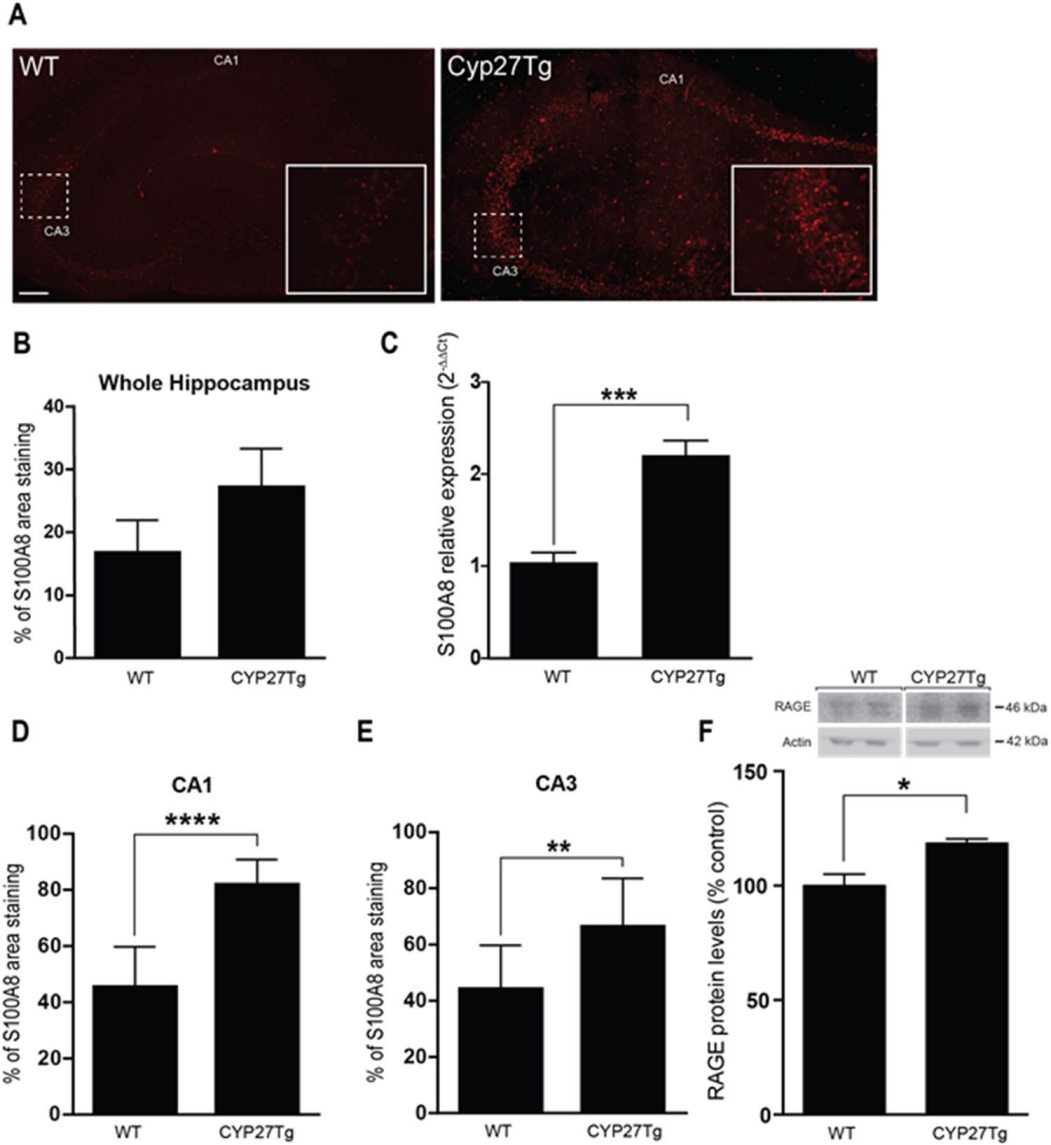
27-OH increases S100A8 and RAGE in CYP27Tg mice. **A** S100A8 immunofluorescence in the hippocampus of CYP27Tg mouse model, overexpressing the human *CYP27A1* gene, versus wild-type mice (12 m.o.) by confocal microscopy. Insets **i** and **ii** show a region in CA3 with S100A8 staining in WT and CYP27Tg, respectively. Bar = 300 µm. **B** Quantification of S100A8 area immuno-stained in whole hippocampi of CYP27Tg mice (*WTmean* = 16.67, *SEM* = 3.038; *CYP27Tgmean* = 27.12, *SEM* = 3.097, *n* = 4 animals per group, *P* = 0.06). **C** Levels of S100A8 mRNA in CYP27Tg hippocampi vs. WT counterparts (12 m.o. *P* < 0.0001, *n* = 4 animals per group). **D** Quantification of S100A8 area immuno-stained in CA1 regions of CYP27Tg mice (*mean* 81.83, *SEM* = 2.84, *P* < 0.0001, *n* = 10 fields from 4 animals). **E** Quantification of S100A8 area immuno-stained in CA3 region of hippocampus of WT (*mean* = 44.16, *SEM* = 5.16, *n* = 9 fields from 4 animals) vs CYP27Tg (*mean* = 66.32, *SEM* = 5.46, *n* = 10 fields from 4 animals, *P* = 0.0094). **F** Protein levels of RAGE in whole lysates from CYP27Tg mice (*mean* = 117.9, *SEM* = 1.64, *n* = 3 animals) vs. WT mice at 12 m.o. (*mean* = 100, *SEM* = 5.57, *n* = 4 animals, *P* = 0.044)

Previously, we have shown that 27-OH mediates the negative effects of dietary cholesterol on several brain function related to cognition impairment [36]. Here, we explore whether the effects observed on S100A8 levels in the HFD mice may also be mediated by 27-OH. In a mouse model overexpressing the human CYP27a1 gene (CYP27Tg), having 5–6 times higher levels of 27-OH than WT animals, the level of S100A8 expression is higher in the hippocampus as shown by immunostaining (Fig. 2A and B) and by qPCR (Fig. 2C), especially in the pyramidal layers of CA1 and CA3 (Fig. 2D and E). When measured by western blot, the expression of RAGE was also increased compared to WT animals (Fig. 2F).

**Fig. 3:**
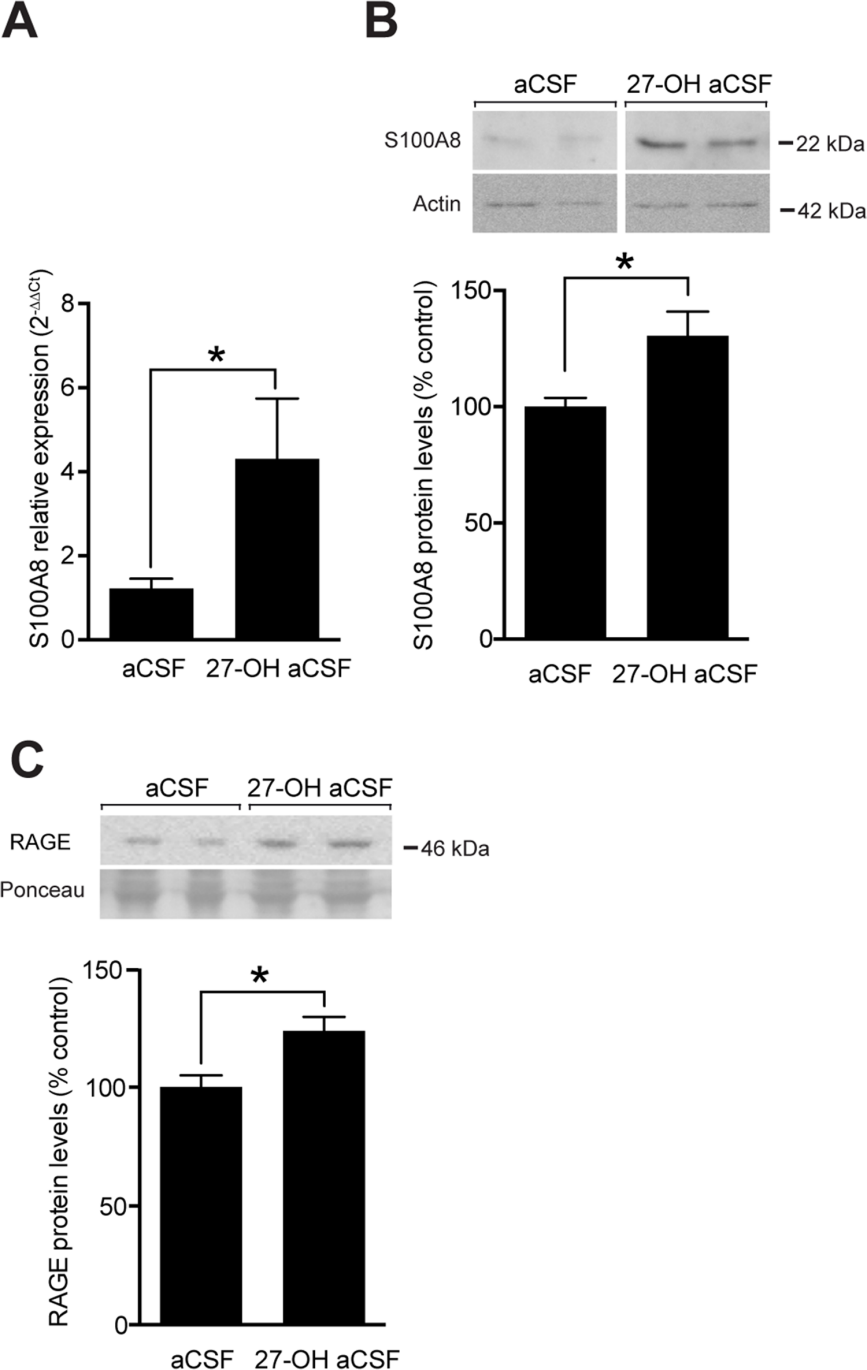
27-OH increases S100A8 and RAGE expressions administered acutely in the brain. Intracerebroventricularly injected 27-OH (ICV27-OH) (10 μM) into the lateral ventricle of wild-type mice showing (**A**) mRNA levels of S100A8 in aCSF controls (*mean* = 1.22, *SEM* = 0.23, *n* = 4 animals, *P* = 0.018) and on 27-OH mice (*mean* = 4.30, *SEM* = 1.43, *n* = 4 animals). Protein levels by western blot of S100A8 (**B**) (*WTmean* = 100, *SEM* = 3.81, *n* = 4 animals; *CYP27Tgmean* = 110.3, *SEM* = 10.3, *n* = 4 animals, *P* = 0.025) and RAGE (**C**) (*WTmean* = 100, *SEM* = 34.97, *n* = 4 animals; *CYP27Tgmean* = 123.8, *SEM* = 5.94, *n* = 4 animals, *P* = 0.021) of ICV27-OH

To confirm that the effects seen in the CYP27Tg mice are the effect of increased 27-OH, we injected 27-OH intracerebroventricular (ICV) into the lateral ventricle of WT mice at a concentration of 10 μM in artificial cerebrospinal fluid (27-OH aCSF). These mice exhibited in addition to the similar increase in the RNA expression of S100A8 (Fig. 3A) higher protein levels (Fig. 3B). As shown in Fig. 3C, RAGE protein levels were elevated in this acute model similarly to CYP27Tg mice.

### Astrocytes Upregulate S100A8 and RAGE Expressions in the Presence of High 27-OH Levels

**Fig. 4:**
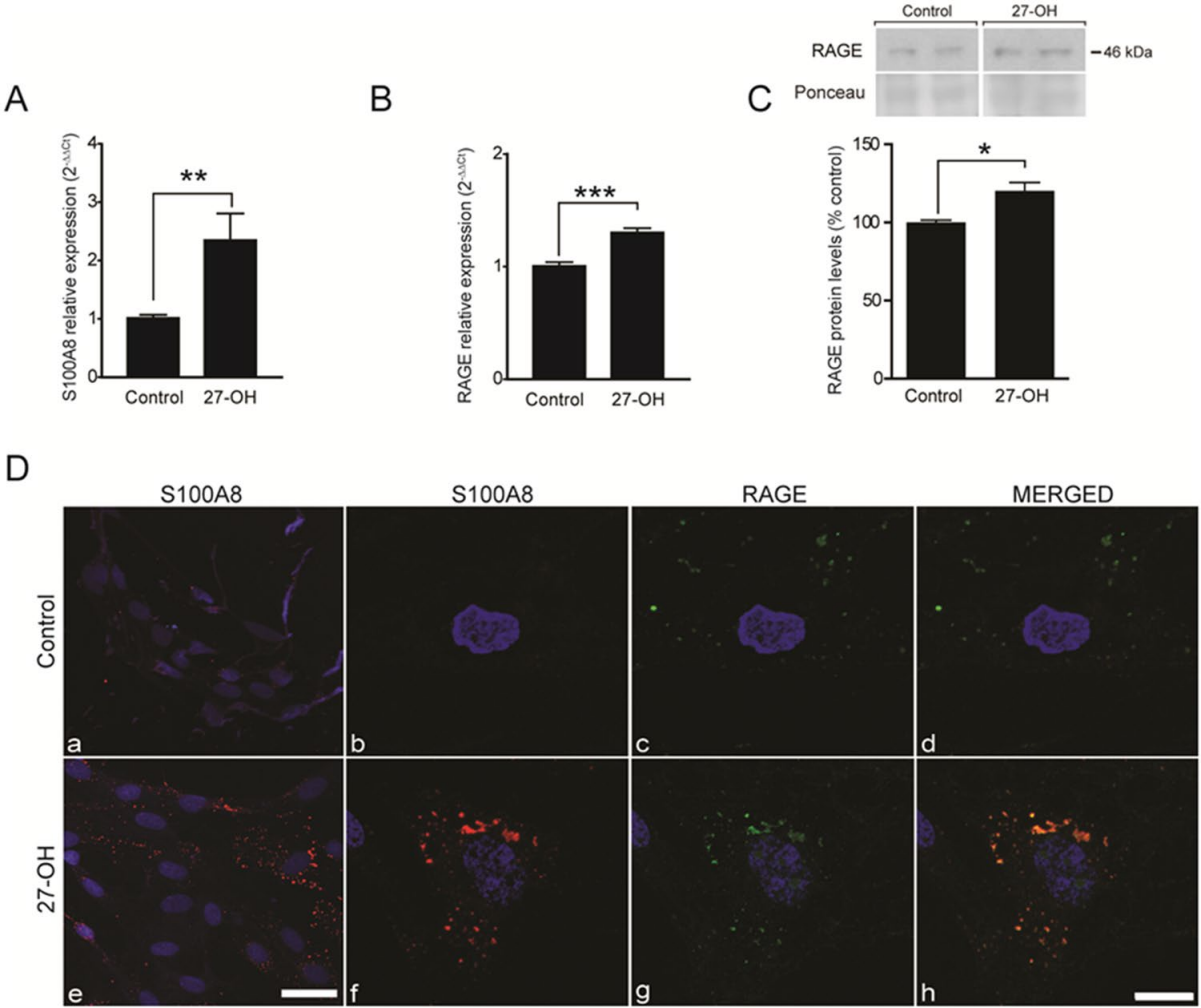
27-OH increases S100A8 and RAGE expressions in astrocytes*. A* Expression levels of S100A8 in glial cells from primary rat cultures treated with vehicle (*CNTmean* = 1.016, *SEM* = 0.05, *n* = 12 wells) or with 27-OH (1 μM, 24 h, *27-OHmean* = 2.350, *SEM* = 0.45, *n* = 12 wells, *P* = 0.008) by RT-qPCR. **B** RAGE mRNA levels in glial cells from primary rat cultures treated with vehicle (*CNTmean* = 1.007, *SEM* = 0.13, *n* = 15 wells) or with 27-OH (1 μM, 24 h, *27-OHmean* = 1.304, *SEM* = 0.037, *n* = 16 wells, *P* < 0.0001). **C** Confocal microscopy of glial cell cultures untreated (upper panel) or treated (lower panel) with 27-OH (1 μM, 24 h) and stained with anti-S100A8 (red), anti- RAGE (green), and DAPI (blue). **a** and **e** are overview images. Scale bar in **e**: 75 µm. For all other panels refer to scale in **h**: 25 µm

Primary rat astrocytic cultures were treated with 27-OH (1 μM, 24 h). Astrocytes produced a significant increase in S100A8 mRNA expression (Fig. 4A). RAGE mRNA (Fig. 4B) and protein (Fig. 4C) levels were increased likewise. In addition, confocal image analysis of these glial cultures treated with 27-OH (1 μM, 24 h) corroborated these results showing apparent increases in S100A8 and RAGE immunoreactivities, in addition to their colocalization (Fig. 4D).

### 27-OH-Induced RAGE Upregulation in Astrocytes Are Mediated by RXRγ

**Fig. 5:**
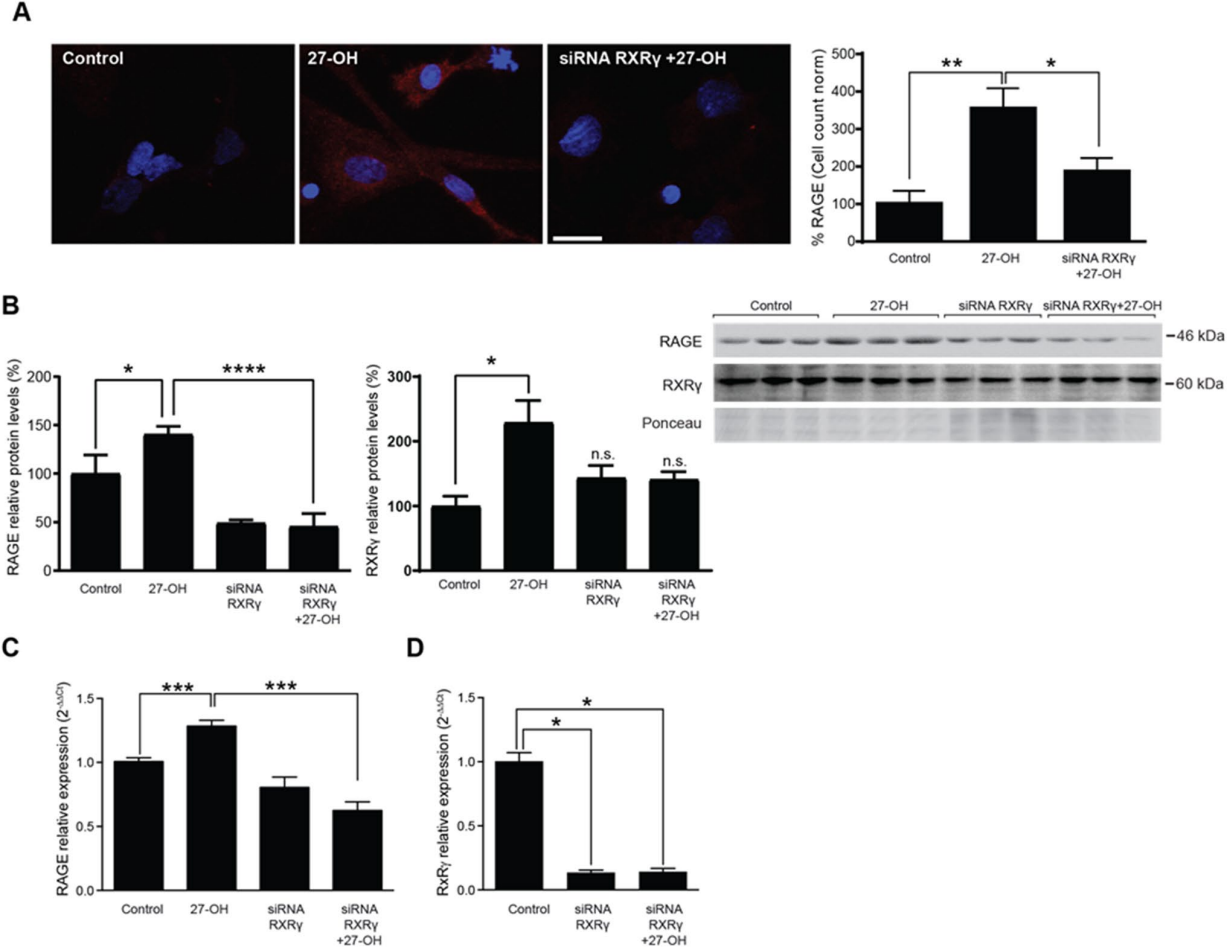
27-OH induction of RAGE in astrocytes is mediated by RXRγ. **A** Knockdown of RXRγ was done using siRNA. RAGE protein levels were increased in astrocytes in culture with 27-OH treatment (1 µM) and prevented with knocking down of RXRγ as observed by immunostaining acquired by confocal imaging (% of area staining normalized to cell number; 1 field per well cultured. *Controlmean* = 100, *SEM* = 20.4, *n* = 3 fields; *27-OHmean* = 353.8, *SEM* = 55.32, *n* = 4 fields; *iRXRg* + *27-OHmean* = 185.9, *SEM* = 18.34, *n* = 4 fields. ANOVA *P* = 0.005. Tukey’s multiple comparisons (*) *P* < 0.05, (**) *P* < 0.001). **B** Knockdown of RXRγ prevented the increase of RAGE mediated by 27-OH (1 µM) at the protein level (*Controlmean* = 100, *SEM* = 11.01, *n* = 3 wells; *27-OHmean* = 140.4, *SEM* = 4.815, *n* = 3 wells; *iRXRgmean* = 49.22, *SEM* = 1.824, *n* = 3wells; *iRxRg* + *27-OHmean* = 45.62, *SEM* = 7.70, *n* = 3wells. ANOVA *P* < 0.0001. Tukey’s multiple comparisons (*) *P* = 0.0173, (****) *P* ≤ 0.0001). RXRγ densitometry shows its increase by 27-OH treatment, which is prevented by siRNA (*Controlmean* = 100, *SEM* = 15.37, *n* = 3 wells; *27-OHmean* = 228.4, *SEM* = 34.37, *n* = 3 wells; *iRXRgmean* = 143.1, *SEM* = 19.27, *n* = 3wells; *iRXRg* + *27-OHmean* = 140.7, *SEM* = 12.09, *n* = 3wells. ANOVA *P* = 0.028. Tukey’s multiple comparisons (*) *P* = 0.02; n.s. *iRXRg* = 0.27; n.s. *iRXRg* + *27-OH* = 0.68). **C** mRNA levels of RAGE were diminished in the RXRγ knockdown astrocytes treated with 27-OH (*Controlmean* = 1.007, *SEM* = 0.03, *n* = 18 wells; *27-OHmean* = 1.284, *SEM* = 0.04, *n* = 19 wells; *iRXRgmean* = 0.804, *SEM* = 0.08, *n* = 6 wells; *iRXRg* + *27-OHmean* = 0.6241, *SEM* = 0.06, *n* = 6 wells. ANOVA *P* < 0.0001. Tukey’s multiple comparisons (***) *P* ≤ .001). **D** RXRγ knockdown prevented its own upregulation by 27-OH (*Controlmean* = 1.004, *SEM* = 0.06, *n* = 3 wells; *iRXRgmean* = 0.1354, *SEM* = 0.019, *n* = 3 wells; *iRXRg* + 27-*OHmean* = 0.14, *SEM* = 0.02, *n* = 3 wells. ANOVA *P* < 0.0001. Tukey’s multiple comparisons (*) *P* < 0.05)

To decipher the possible mechanism through which 27-OH increases S100A8 and RAGE in astrocytes, we tested the role of the RXRγ, which we previously found mediates detrimental effects of 27-OH on neuron function [37, 38]. RXRγ is also expressed in hippocampal astrocytes, has been implicated in regulation of cholesterol metabolism, and has been suggested previously as a pharmacological target for AD [39–41]. Therefore, we decided to probe RXRγ as a potential mediator of the effects of 27-OH on RAGE expression in astrocytes. RXRγ was knocked down in astrocytes using siRNA and treatments with 27-OH. As seen by immunohistochemistry in Fig. 5A, the effect of high 27-OH on RAGE protein levels in astrocytes was decreased when combined with the RXRγ knockdown. Likewise, RXRγ knockdown blocked the 27-OH-induced RAGE increase of protein levels (Fig. 5B), as well as mRNA levels (Fig. 5C), with a knockdown efficiency of RXRγ of around 80% of control levels (Fig. 5D).

### 27-OH-Induced RAGE Increase in Neurons Is Mediated by RXRγ and Not by Direct LXRβ Activation

**Fig. 6:**
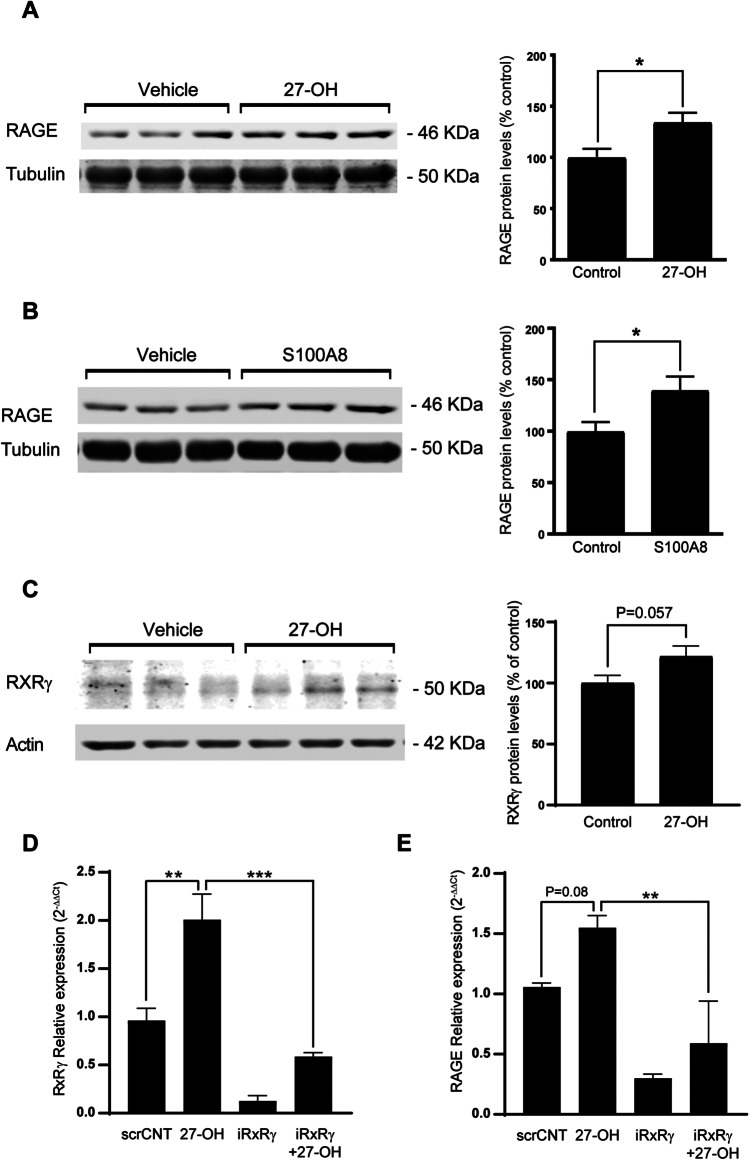
27-OH-induced RAGE increase in neurons. **A** Western blots from rat primary neurons treated with 27-OH (1 μM, 24 h) showing protein levels of RAGE (*Vehiclemean* = 1.0, *SEM* = 0.08, *n* = 8 wells; *27-OHmean* = 1.34, *SEM* = 0.09, *n* = 8 wells. Unpaired *t* test *P* = 0.01). **B** Western blots from rat primary cultures treated with S100A8 (12.5 ug/ml, 24 h) showing protein levels of RAGE (*Vehiclemean* = 1.0, *SEM* = 0.08, *n* = 12 wells; *27-OHmean* = 1.39, *SEM* = 0.13, *n* = 12 wells. Unpaired *t* test *P* = 0.02). **C** Western blots from rat primary neurons treated with 27-OH (1 μM, 24 h) showing protein levels of RXRγ (*Vehiclemean* = 1.0, *SEM* = 0.08, *n* = 8 wells; *27-OHmean* = 1.34, *SEM* = 0.09, *n* = 8 wells. Unpaired *t* test *P* = 0.053) and **D** mRNA levels of RXRγ were diminished in the RXRγ-knockdown primary neurons treated with 27-OH (*Controlmean* = 0.96, *SEM* = 0.1247, *n* = 4 wells; *27-OHmean* = 2.007, *SEM* = 0.26, *n* = 6 wells; *iRXRgmean* = 0.12, SEM = 0.056, *n* = 4 wells; *iRXRg* + *27-OHmean* = 0.58, *SEM* = 0.04, *n* = 4 wells. ANOVA *P* < 0.0001. Tukey’s multiple comparisons (**) *P* = 0.005; (***) *P* = 0.002), and a similar effect was found on RAGE mRNA expression (*Controlmean* = 1.057, *SEM* = 0.033, *n* = 4 wells; *27-OHmean* = 1.54, *SEM* = 0.101, *n* = 4 wells; *iRXRgmean* = 0.29, *SEM* = 0.035, *n* = 4 wells; *iRXRg* + *27-OHmean* = 0.59, *SEM* = 0.35, *n* = 4 wells. ANOVA *P* = 0.0003. Tukey’s multiple comparisons (**) *P* = 0.0045)

To test if high 27-OH levels were affecting RAGE expression in neurons, we used rat primary cortico-hippocampal cultures. When mature neurons were treated with 27-OH 1 µM for 24 h, we observed again an increase in RAGE protein levels compared to vehicle (Fig. 6A). Additionally, treatments with recombinant murine S100A8 12.5 µg/ml for 24 h showed significant increased RAGE protein levels (Fig. 6B). We previously reported 27-OH induces overexpression of RXRγ in neurons during differentiation; here, we treated neurons after 10 DIV for 24 h with 1 µM of 27-OH, and we found no change of RXRγ levels by western blot (Fig. 6C) (*P* = 0.057). When we knocked down RXRγ using siRNA, we found that RAGE increase with 27-OH was prevented (Fig. 6D). When LXRs were blocked using 22(S)-OH treatments, RAGE increase is not prevented in the presence of high 27-OH levels (Fig. S4), which suggest LXRβ activation does not directly induces RAGE expression. Treatments with high 27-OH levels did not induce significant changes on the total levels of NFk-B (Fig. S6A) in a similar way as S100A8 did (Fig. S6B); nevertheless, NFk-B nuclear translocation was observed in the human neuroblastoma cell line SH-SY5Y (Fig. 6C).

## Discussion

Here, we report that excessive 27-OH triggers an alarmin response involving S100A8 and its receptor RAGE in both neurons and astrocytes, in vivo and in vitro. These results are consistent to the hypothesis proposing that 27-OH mediates detrimental effects of high-cholesterol in the plasma over the central nervous system (CNS), as shown by our experiments with HFD mice and in congruence with previous works from our group [15, 16, 34, 38]. While more evidence exists of the effect of 27-OH in neurons, our findings propose an astrocytic response to 27-OH involving the S100A8 alarmin.

In the periphery, S100A8 is involved in pathological cascades in multiple diseases such as rheumatoid arthritis [42], systemic erythematosus lupus [43], and cancer [44]. In the CNS, S100A8 is related to neurodegenerative diseases such as AD, where the expression of S100A8 is twofold higher in the hippocampi of AD patients compared to that of non-demented cases [45]. The same is suspected to happen in other neurodegenerative diseases like postoperative cognitive dysfunction [46] and through autophagy alterations in Parkinson’s disease [47]. Moreover, treatment with Aβ increased the expression of S100A8 in different paradigms of glial cultures: by 30-fold in microglia chronic treatment after oligomeric Aβ increased the expression of S100A8 [48] and by twofold in astrocytes after Aβ_42_ incubation for only 24 h [23]. Previously, we also observed that S100A8 treatments increased the levels of Aβ_42_ suggesting a positive feedback between both their productions [23]. The intracellular colocalization of RAGE and S100A8 in cultured astrocytes shown in our work suggest a possible response to acute 27-OH treatments through internalization, while we have seen extracellular accumulation of S100A8 aggregates in CYP27Tg brains (data not shown); this could suggest that vesicular traffic would be important in the regulation of S100A8/RAGE signaling, and further experiments should be done to explore this avenue.

Our results show that in vivo, both CYP27Tg and the ICV27-OH mouse models increased their levels of S100A8 and RAGE in the brain as a response to excessive 27-OH levels. RAGE expression in the CNS promotes neurite outgrowth and neuronal differentiation [26] and participates in repairing injured nerves [49]. However, chronic RAGE stimulation affects neuronal function promoting both Tau phosphorylation and Aβ production, which would result in synaptic dysfunction and neurodegeneration [50]. Furthermore, the hippocampus of AD patients shows enhanced expressions of RAGE, Aβ, and advanced glycation end-products (AGE) as S100A8 [51]. Indeed, the increase in RAGE levels found in AD was shown in neurons, astrocytes, microglia, and endothelial cells [52, 53], and it is suggested to contribute to mechanism of AD pathogenesis such us oxidative stress, inflammation, neuronal dysfunction, and impairment of short-term memory [54–56].

Data from single-cell sequencing shows that S100A8 is not expressed by hippocampal neurons (Figure S3); however, it is prominently expressed in the mouse brain [57], so it must be in glial cells mediating the alarmin response such as astrocytes, as seen here by immunocytochemistry. This can be further confirmed consulting AD-oriented databases on single-cell expression such as scREAD, which shows microglia and astrocyte overexpressing S100A8 compared to WT mice in several datasets [58]. Given the low expression of neuronal S100A8, the effects of 27-OH over alarmin signaling in neurons is minimal; nevertheless, the induction of S100A8 and RAGE on astrocytes possibly contributes significantly to sterile inflammation. Together with other known neuronal effects [38, 59], high 27-OH levels in the brain can be detrimental for overall brain function due to several cascades that might potentiate between them. In this regard, patients with hereditary spastic paraplegia type 5A (SPG5) have very high 27-OH levels in their brain due to a mutation in the gene CYP7B1 encoding oxysterol-7α-hydroxylase [60]. While 27-OH has been found to be neurotoxic, SPG5 patients do not show significant neurodegeneration, which suggest that the interaction between different brain cell types could produce additional metabolites that could play a neuroprotective effect. On the other hand, macrophage activation induces a significant elevation of 25-OH in plasma upon activation [61], but whether this can be translated to microglial activation remains unknown to us. Also, since the CYP27Tg model has a normal CYP7B1 gene; it is possible that some of the in vivo effects here described could be mediated by intermediates of 27-OH metabolism like cholestenoic acids or 25-OH, since 27-OH has a rapid catabolism in the brain [62].

**Fig. 7:**
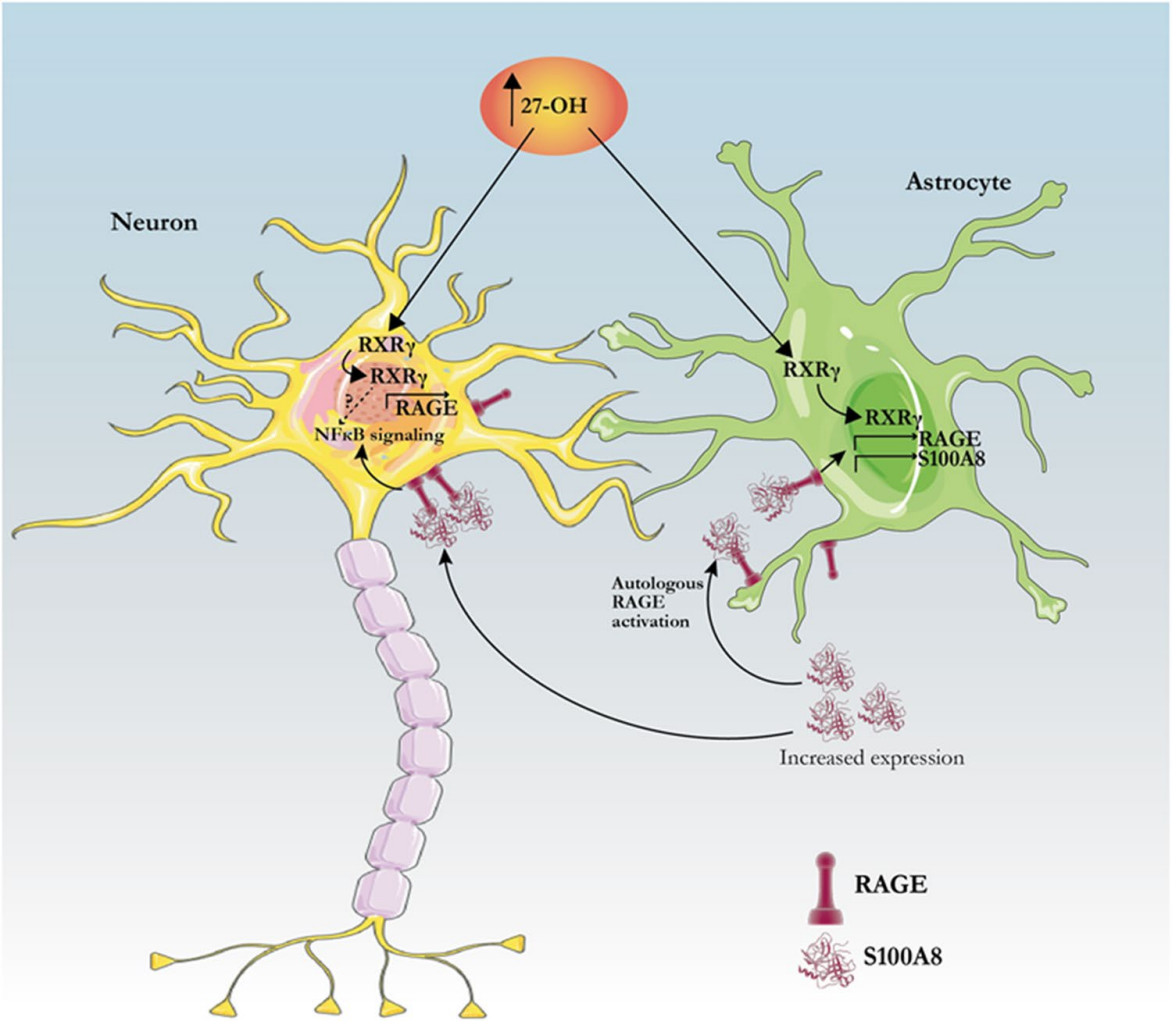
Proposed mechanism of alarmin induction by 27-OH. Elevated levels of 27-OH in the brain activate RXRγ in astrocytes, which induce expression of both RAGE and S100A8. S100A8 can then signal autologously astrocytic RAGE and further induce its expression, possibly inducing internalization of S100A8/RAGE complexes and activating astrocyte RAGE signaling for sterile inflammation. Astrocytic S100A8 activates neuronal RAGE as well, inducing alarmin signaling through NFk-B. In parallel, 27-OH can activate neuronal RXRγ inducing RAGE expression, which further sensitizes neurons to the binding of S100A8. It is unclear whether RXRγ activation by 27-OH contributes to alarmin signaling on its own (dashed arrow and question mark in the figure)

We previously found RXRγ is a mediator of 27-OH detrimental effects in neurons [38]; however, whether RXRγ played a role in astrocytes was unknown. siRNA silencing of RXRγ eliminated the 27-OH-induced increase of RAGE levels in astrocytes as seen by immunohistochemistry, western blot, and qPCR. RXRγ is a nuclear receptor that dimerizes with LXRs and mediates transcription of genes involved in cholesterol and lipid metabolism [63–66]. Broad LXR blocking in astrocytes did not prevent RAGE increase mediated by 27-OH (Fig. S5). It is possible that 27-OH binds directly to RXRγ or promotes its dimerization with LXR or another nuclear receptor such as RORα, which have been shown to bind oxysterols [67]. We showed that a similar mechanism regulating RAGE expression is present in neurons, since knockdown of RXRγ prevented 27-OH-induced RAGE expression at the mRNA level. Also, 27-OH induction of RAGE in primary neurons was independent of the presence of S100A8, meaning that high levels of 27-OH can sensitize neurons to alarmin signaling by increasing the amount of RAGE receptors before inflammation takes place (Fig. 7). This becomes evident when treating a human neuroblastoma cell line with high levels of 27-OH, which induces nuclear translocation of NFk-B (Fig. S5). Finally, we propose that the effect of 27-OH over neuronal alarmin cascades can take place in humans since similar effects are found on human neuroblastoma cells (Fig. S1 and Fig. S5).

## Conclusions

Our data supports the notion that high levels of peripheral cholesterol generate in turn increased 27-OH levels and enhance an inflammatory signaling cascade in the brain. HFD and excessive 27-OH result in S100A8 accumulation and increased RAGE expression in neurons and astrocytes and therefore enhance alarmin cascades mediated by RXRγ [20, 23]. These results place 27-OH as a mediator of the pathological mechanisms linking hypercholesterolemia and sterile inflammation in the brain, with potential implication in the pathophysiology of AD and other neurodegenerative diseases.

## Supplementary Information

Below is the link to the electronic supplementary material.

Supplementary file1 (DOC 1943 kb)

## Data Availability

Data for this work is archived and publicly available upon request to the corresponding authors, as well as at the Karolinska Institutet repository and the Swedish National Archive.

## Code Availability

Software used in this work is commercially available. No custom coding was done for this work.

## Abbreviations

AD: Alzheimer’s disease
APOEε4: Apolipoprotein E, allele epsilon 4
ApoJ: Clusterin
BBB: Blood–brain barrier
27-OH: 27-Hydroxycholesterol
CYP27Tg: CYP27A1 overexpressor
CSF: Cerebrospinal fluid
RAGE: Receptor for advanced glycation end product
RXRγ: Retinoid X receptor gamma
HFD: High-fat/high-cholesterol diet
WT-HFD: Wild-type mice (WT) fed with HFD
WT-ND: Wild-type mice (WT) fed with normal diet
CA: Corpora amylacea
ICV: Intracerebroventricular injections
aCSF: Artificial cerebrospinal fluid
CNS: Central nervous system
HDL: High-density lipoprotein
NFk-B: Nuclear factor kappa-light-chain-enhancer of activated B cells
SPG5: Hereditary spastic paraplegia type 5A
