# Supplementary material for: Hypercholesterolemia and 27-Hydroxycholesterol Increase S100A8 and RAGE Expression in the Brain: a Link Between Cholesterol, Alarmins, and Neurodegeneration
Source: Mol Neurobiol. 2021 Aug 27;58(12):6063–76. doi: 10.1007/s12035-021-02521-8 (PMC8639576; doi:10.1007/s12035-021-02521-8)
Supplement: Supplementary file 1 — Supplementary file1 (DOC 1943 kb) [file 12035_2021_2521_MOESM1_ESM.doc]

**Hypercholesterolemia and 27-hydroxycholesterol increase S100A8 and RAGE expression in the brain: a link between cholesterol, alarmins and neurodegeneration.**

Raúl Loera-Valencia^a^*, Muhammad-Al-Mustafa Ismail^a^*, Julen Goikolea^a^, Maria Lodeiro^a^, Laura Mateos^a^, Ingemar Björkhem^b^, Elena Puerta^a,c^, Mariana A. Romão^e,f^, Cláudio M. Gomes^e,f^, Paula Merino-Serrais^a,g,h^, Silvia Maioli^a^ and Angel Cedazo-Minguez^a^*.

^a^ Karolinska Institutet, Center for Alzheimer Research, Department of Neurobiology Care Sciences and Society, Division of Neurogeriatrics, Stockholm, Sweden.

^b^ Department of Laboratory Medicine, Division of Clinical Chemistry, Karolinska

University Hospital, Huddinge, Sweden.

^c^ Department of Pharmacology and Toxicology, University of Navarra, Pamplona, Spain.

^e^ Biosystems and Integrative Sciences Institute, Faculdade de Ciências, Universidade de Lisboa, 1749-016 Lisboa, Portugal.

^f^ Departamento de Química e Bioquímica, Faculdade de Ciências, Universidade de Lisboa, 1749-016 Lisboa, Portugal.

^g^ Laboratorio Cajal de Circuitos Corticales (CTB), Universidad Politécnica de Madrid, Madrid, Spain.

^h^ Departamento de Neurobiología Funcional y de Sistemas, Instituto Cajal, CSIC, Madrid, Spain.

^*^Equal contribution.

Correspondence to:

Ángel Cedazo-Mínguez,

[Angel.Cedazo-Minguez@ki.se](mailto:Angel.Cedazo-Minguez@ki.se)

and

Raúl Loera-Valencia,

[Raul.loera@ki.se](mailto:Raul.loera@ki.se)

Names and emails of authors:

Raúl Loera-Valencia

Raul.loera@ki.se

Muhammad-Al-Mustafa Ismail

muhammadalmustafa@gmail.com

Julen Goikolea

Julen.goikolea@ki.se

Maria Lodeiro

Maria.lodeiro@ki.se

Laura Mateos

Laura.mateos@ki.se

Ingemar Björkhem

Ingemar.bjorkhem@ki.se

Elena Puerta

epuerta@unav.es

Mariana A. Romão

maromao@fc.ul.pt

Cláudio M. Gomes

cmgomes@fc.ul.pt

Paula Merino-Serrais

paula.merino-serrais@cajal.csic.es

Silvia Maioli

Silvia.maioli@ki.se

Angel Cedazo-Minguez

Angel.cedazo-minguez@ki.se

**ABSTRACT**

Alterations in cholesterol metabolism in the brain have a major role in the physiology of Alzheimer´s disease (AD). Oxysterols are cholesterol metabolites with multiple implications in memory functions and in neurodegeneration. Previous studies have shown detrimental effects of cholesterol metabolites in neurons, but its effect in glial cells is unknown. We used a high fat/high cholesterol diet in mice to study the effects of hypercholesterolemia over the alarmin S100A8 cascade in the hippocampus. Using CYP27Tg, a transgenic mouse model, we show that the hypercholesterolemia influence on the brain is mediated by excess of 27-hydroxycholesterol (27-OH), a cholesterol metabolite. We also employed an acute model of 27-OH intraventricular injection in the brain to study RAGE and S100A8 response. We used primary cultures of neurons and astrocytes to study the effect of high levels of 27-OH over the S100A8 alarmin cascade. We report that a high fat/high cholesterol diet leads to an increase in S100A8 production in the brain. In CYP27Tg we report an increase of S100A8 and its receptor RAGE in the hippocampus under elevated 27-OH in the brain. Using siRNA, we found that 27-OH upregulation of RAGE in astrocytes and neurons is mediated by the nuclear receptor RXRγ. Silencing RXRγ in neurons prevented 27-OH mediated upregulation of RAGE. These results show that S100A8 alarmin and RAGE respond to high levels of 27-OH in the brain in both neurons and astrocytes through RXRγ. Our study supports the notion that 27-OH mediates detrimental effects of hypercholesterolemia to the brain via alarmin signaling.

**KEYWORDS:** Alarmins, Astrocytes, Retinoid receptors, Oxysterols, Alzheimer’s disease, sterile inflammation.

**BACKGROUND.**

Alzheimer’s disease (AD) is strongly associated with elevated circulating cholesterol during mid-life (1-5). GWAS have identified risk genes involved in cholesterol metabolism in the brain including Apolipoprotein E, allele epsilon 4 (APOEε4), Clusterin (ApoJ) and the ATP Binding Cassette Subfamily A Member 7 (ABCA7) (6-9). Nevertheless, cholesterol itself does not cross the blood brain barrier (BBB), posing the question of how plasma hypercholesterolemia is linked to the risk of developing AD. Moreover, cholesterol lowering therapies do not have a clear effect improving cognition of AD patients (10-12). Unlike cholesterol, its side-chain oxidized metabolite, 27-hydroxycholesterol (27-OH), is able to traverse the BBB and correlates directly to circulating cholesterol levels in plasma in humans (13).

We have reported alterations in cognition observed in mice on a high fat diet (HFD) (14) as well as in the chronically high 27-OH transgenic mice, CYP27A1 overexpressor (CYP27Tg) (15), suggesting that 27-OH may be mediate some of the deleterious effects of high peripheric cholesterol in the brain (16). In support of this, we have shown that 27-OH affects the levels of the activity-regulated cytoskeleton-associated protein (Arc), a protein involved in long-term consolidation of memory (16).

27-OH have a proinflammatory role in atherosclerosis, being the most abundant oxysterol in atherosclerotic plaques (17). Whether 27-OH mediates inflammation in the brain is yet not known, however, AD patients exhibit higher levels of this oxysterol in their brains and cerebrospinal fluid (CSF) (18) and inflammation is considered an important feature of AD (19).

Sterile inflammation describes a response not triggered by microbial agents, but by endogenous molecules called alarmins that are released during tissue damage (20). A number of alarmins are increased in the AD brain, including S100 calcium-binding proteins (21). We have recently reported that there is a positive feedback between Aβ and S100A8 productions in brain cells and that mice overexpressing the precursor of Aβ, accumulate S100A8 aggregates in the brain prior to the appearance of Aβ plaques (22). Furthermore, S100A8 is one of the ligands of the Receptor for Advanced Glycation End product (RAGE) (23), expressed in almost all brain cells including microglia, neurons and astrocytes (24, 25). Activation of RAGE is involved in inflammatory responses, promoting synaptic dysfunction and neurodegeneration (26).

Here, we investigate the effects of a high cholesterol diet and 27-OH on S100A8 and RAGE in the brain. Utilizing several *in vivo* models, primary cultures of glial cells and neurons treated with 27-OH, we report that 27-OH increases S100A8 and RAGE levels in the brain. Most interestingly, 27-OH mediates these effects involving the Retinoid X Receptor gamma (RXRγ) receptor in astrocytes and neurons, while activation of the Nuclear Factor kappa-light-chain-enhancer of activated B cells (NFk-B) is also shown.

**METHODS.**

**Animals**

Five-to-six weeks-old (C57BL/6) mice were obtained from B&K (Sollentuna, Sweden). The mice were grouped based on diet into two groups, normal chow diet (ND) or a high-fat diet (HFD) containing 21% fat and 0.15% cholesterol (R638, Lactamine, Sweden) for 9 months. Generation and breeding of the CYP27Tg mice have been described previously (27). These mice were kept with food and water *ad libitum* for 12 months before sacrifice. Wild-type C57BL/6 mice were purchased from Charles River (Germany). These mice were stereotaxically injected with 1 μl of 27-OH into the lateral ventricle of both hemispheres, more details can be found below.

All mice were kept under controlled conditions of humidity and temperature on a 12-h light-dark cycle. Food and water were provided ad libitum. Animals were sacrificed by decapitation and the brains immediately frozen on dry ice and stored at −80 °C Experimental procedures involving animals were conducted in accordance with the European regulation and approved by the Swedish Board of Agriculture (ethical permits ID S33-13, extension 57-15 and 4884/2019). All efforts were made to minimize suffering or distress to experimental animals.

**Stereotaxic injections**

C57BL/6 mice were anesthetized with an isoflurane/oxygen mixture while being fixated on a stereotaxic frame (David Kopf Instruments) and placed on a heated pad at 37°C to maintain normal body temperature. The lateral ventricle was injected bilaterally with 1 μl of solution per injection site, using a Hamilton syringe (10 μl gauge). The coordinates were set according to the Paxinos Mouse brain atlas and were 2.0 mm from the skull surface, -0.9 mm anteroposterior, ± 1.4 mm laterally from the bregma. The injection solution is composed of high-density lipoprotein (HDL) associated to 27-OH diluted in artificial CSF (aCSF) (RD Systems, 3525) and used at a final concentration of 10 μM. HDL diluted in aCSF was used for the control group. Incubation of 27-OH at a concentration ratio of 1:3 with HDL (Abcam, ab77881) at 37°C for 1 h was done to produce the HDL-associated 27-OH.

**Cell cultures and treatments**

Cerebellar tissue from 18-day-old Sprague–Dawley rat embryos was dissected. Cerebellar tissue was dissociated and seeded in Dulbecco’s Modified Eagle Medium (DMEM/F12) (Life Technologies, CA, USA) supplemented with 10% inactivated fetal bovine serum (FBS) (Life Technologies, CA, USA) in T75 plastic culture flasks (Corning, NY, USA). Cultures were incubated at 37°C, 95% air /5% CO_2_ and culture media were replaced biweekly. Inactivated astrocyte dominated cultures at 10-14 days were used as previously identified by immuoncytochemical characterization (28). 27-OH was obtained from Steraloids (Newport, Rhode Island, USA). Treatments were done after a 24 h period of being cultured in serum-free media and treated with 27-OH at a concentration of 1 μM for 24 h. Preincubation with 22(S)-Hydroxycholesterol (22(S)-OH, 10 μM), a liver-X-receptor (LXR) inhibitor, was 3 h. Murine S100A8 was expressed in *E. coli* BL21(DE3) and purified to homogeneity using previously established protocols (29, 30) and quantitated using reported extinction coefficients (31). Ethical consent for experiments with primary cultures was received from the regional ethical committee of Karolinska Institutet and by the the Swedish Board of Agriculture (ethical permits ID S33-13, extension 57-15 and 4884/2019).

Human SH-SY5Y (ATCC® CRL-2266™) neuroblastoma cells were obtained from the American Type Culture Collection (ATCC) and cultured in a 1:1 mixture of DMEM/F-12 media and supplemented with 10% fetal bovine serum in T75 plastic culture flasks (Corning, NY, USA) at 37ºC, with 5% CO2. For treatments, cells were seeded in 6 well plates and when confluent treated with 27-OH at 10 µM for 24h. The nuclear and cytoplasmic extraction reagents kit NE-PER (Pierce, Rockford, IL, USA) was used to isolate the nuclear and cytosolic fractions of SH-SY5Y cells following the manufacturer’s protocol. Protease inhibitor cocktail (1:500, Sigma-Aldrich) was added freshly. To quantify, immunoblotting with antibody against the nuclear marker Lamin-A/C was performed (Sigma-Aldrich SAB4200236,1:1000).

**Small interference RNA transfection**

*Astrocytes.* RXRγ knockdown was performed using siRNA designed by Dharmacon (ON-TARGET plus SMARTpool; L-083061-02-0020). Rat primary astrocytes (80% confluence) were transfected with a final concentration of 25nM per well and 4 µl of DharmaFECT 3 reagent (Dharmacon) according to the manufacturer’s instructions. The efficiency of the knockdown was evaluated using mRNA levels of RXRγ.

*Neurons.* The same siRNA reagent was used for RXRγ knockdown in neurons (ON-TARGET plus SMARTpool; L-083061-02-0020). Rat primary neurons (80% confluence) at 5DIV were transfected with 25nM per well and 4 µl of DharmaFECT 3 reagent (Dharmacon) according to the manufacturer’s instructions. After 72 hours, the media was replaced for media containing the different treatments of vehicle and 27-OH 1 µM, that remained 24 hours before collection. The efficiency of the knockdown was evaluated using mRNA levels of RXRγ.

**RNA extraction and Real time RT-PCR**

RNA extraction and real-time PCR were performed as previously described (32). Briefly, total RNA was extracted using the RNeasy lipid tissue mini kit from Qiagen (Palo Alto, CA, USA) following the manufacturer´s instructions. Real-time PCR amplification assay for target genes were performed with a total volume of 20 μl in each well containing 10 μl of PCR Master Mix (Life Technologies, CA, USA), 2 μl of cDNA corresponding to 10 ng of RNA, and 1 μl of each TaqMan Gene Expression Assays. Relative quantification of the target genes were done using the comparative cycle threshold method, 2^-ΔΔCt^, where ΔΔCt = (*Ct* _target gene_ – *Ct* _GAPDH_)_treated_ –(*Ct* _target gene_ – *Ct* _GAPDH_)_untreated_. After the 2^-ΔΔCt^ calculations of cDNA for every sample in triplicates expression was portrayed as a mean ± SEM.

**Immunochemistry**

Immunohistochemistry was performed on coronal sections of fresh-frozen brains from the hippocampus of the wild-type ND and HFD mice. The sections were fixed in cold 4% paraformaldehyde (PFA) in saline phosphate buffer (PBS, 0.1M, pH 7.4) for 15 minutes and subsequently washed three times with PBS. After fixation, single immunochemistry was performed. All slices were blocked for 1 h in PBS with 0.25% Triton-X and 3% BSA. The sections were incubated overnight with the primary antibody goat anti-S100A8 (1:100; sc-48352. Santa Cruz) and then for 2 hours at room temperature with the secondary antibody Alexa fluor 594 donkey anti-goat. 4,6 diamidino-2-phenylindole (DAPI) (Sigma, St. Louis, MO, USA) was used to identify the nuclei of cell bodies. Finally, the sections were rinsed in PBS and mounted using the fluorescence mounting medium ProLong Gold Antifade Reagent (Invitrogen Corporation, Carlsbad, CA, USA). The sections were thoroughly washed in PBS throughout the various stages. The primary antibody was omitted with regards to the negative control.

Immunocytochemistry was performed on glial cells from primary cultures. The cells were seeded at 50% confluence onto cover slips. Following 24 h in serum-free media, the treatments were done and then cells were pre-fixed with 2% PFA for 2 minutes and fixed with 4% PFA for 20 minutes. Afterwards, cells were washed three times with PBS. All cover slips were blocked for 30 minutes in PBS with 0.1% Triton-X and 1% BSA. The primary antibodies used were goat anti-S100A8 (1:100; sc-48352, Santa Cruz) and rabbit anti-RAGE (1:100; ab3611, Abcam). The secondary antibodies used were Alexa fluor 594 donkey anti-goat and Alexa fluor 488 chicken anti-rabbit, respectively. Cover slips were first incubated overnight with the primary antibodies and then for 30 minutes at room temperature with secondary antibodies and DAPI to identify the nuclei of cell bodies. Finally rinsed in PBS, the cover slips were mounted as described above. Between the different steps the cover slips were thoroughly washed in PBS. Again, omission of the primary antibody indicates the negative control.

Confocal imaging was performed with a Zeiss LSM 510 META confocal laser scanning system. The ﬂuorescence of DAPI, Alexa 594 and Alexa 488 were recorded through separate channels with either a Plan Apo 40x dry (NA, 0.95) or 63x oil (NA, 1.3) lenses.

**Cresyl violet staining**

Fast-cresyl violet staining was done to visualize the granules found in the *corpora amylacea* in the same sections used for immunohistochemistry. In brief, after the image processing, the sections were stained for 10 minutes with cresyl violet solution. Following staining, the sections were dehydrated and cleared with xylene. Images were taken with an optical microscope (Nikon eclipse E800M) at 20x (NA, 0.75) under bright field optics.

**Western blot and densitometry analysis**

Immunoblotting was carried out in mice hippocampal tissues, primary rat culture in addition to the neuroblastoma cell line. Tissue homogenization and immunoblotting were performed as previously described (33). The following primary antibodies were used: goat anti-S100A8 (sc-48352, Santa Cruz), rabbit anti-RAGE (ab3611, Abcam), rabbit anti-RXRγ (ab15518, Abcam), rabbit anti- NFk-B (PA1-186, Thermo Fischer), rabbit anti-actin (A2103, Sigma) and mouse anti-Lamin AC (SAB4200236, Sigma). Primary antibody incubation was followed by the incubation with the respective secondary immunoglobulin G (IgG) at 1:3000-5000 dilutions (Amersham Biosciences). Immunoreactivity was detected using the ECL detection system (Amersham Biosciences). The relative density of the immunoreactive bands was calculated from the optical density multiplied by the area of the selected band using the ImageJ 1.383 software (NIH, MA).

**Statistical analysis**

Data values are expressed as mean ± standard error mean (SEM). One-way ANOVA was used to compare differences between mean levels of variables among different groups, followed by Tukey’s Post-Hoc test to compare means of every group between each other; or Dunnett’s multiple comparisons to compare to the mean of a control group. Multiplicity adjusted P values are shown for multiple comparisons. Otherwise, unpaired t-test or Main Whitney test was used. A P value of less than 0.05 was considered statistically significant.

**RESULTS**

**High fat/high cholesterol diet enhances the expression of S100A8 in the brain.**

S100A8 expression was analyzed in the hippocampus of wild-type mice (WT) fed with HFD (WT-HFD) compared to a normal diet (WT-ND) through immunohistochemical analysis. Figure 1**A** shows an increase in S100A8 immunoreactivity in the brain of WT-HFD compared to WT-ND (insets a and b). This increase appears as extracellular granular aggregates in the *stratum oriens* and *stratum radiatum* of the hippocampus (Fig. 1**A**, inset *b*), areas of importance for learning and memory processes.

Members of the S100 protein family tend to aggregate and be part of the corpora amylacea (CA), which is found during the course of normal aging in the human brain (34). Consistent with the accumulation of S100A8 seen in AD mouse models (22), cresyl violet staining revealed no overlap between the CA and the S100A8 aggregates (Fig. 1**A**, insets *b* vs. *c*).

Real-time PCR analysis of hippocampal samples from WT-HFD also showed an increase in S100A8 expression compared to WT-ND (Fig. 1**B**). By western blot, we confirmed that HFD induced increase of S100A8 in the hippocampus of mice (Fig. 1**C**).

**27-OH increases S100A8 and RAGE expression *in vivo.***

Previously, we have shown that 27-OH mediates the negative effects of dietary cholesterol on several brain function related to cognition impairment (35). Here we explore whether the effects observed on S100A8 levels in the HFD mice may also be mediated by 27-OH. In a mouse model overexpressing the human CYP27a1 gene (CYP27Tg), having 5-6 times higher levels of 27-OH than WT animals, the level of S100A8 expression is higher in the hippocampus as shown by immunostaining (Fig. 2**A** and **B**) and by qPCR (Fig. 2**C**), especially in the pyramidal layers of CA1 and CA3 (Fig. 2**D** and **E**). When measured by western blot, the expression of RAGE was also increased compared to WT animals (Fig. 2**F**).

To confirm that the effects seen in the CYP27Tg mice are the effect of increased 27-OH, we injected 27-OH intracerebroventricularly (ICV) into the lateral ventricle of wild-type mice at a concentration of 10 μM in artificial cerebrospinal fluid (27-OH aCSF). These mice exhibited in addition to the similar increase in the RNA expression of S100A8 (Fig. 3**A**), higher protein levels (Fig. 3**B**). As shown in Figure 3**C**, RAGE protein levels were elevated in this acute model similarly to CYP27Tg mice.

**Astrocytes upregulate S100A8 and RAGE expressions in the presence of high 27-OH levels.**

Primary rat astrocytic cultures were treated with 27-OH (1 μM, 24 h). Astrocytes produced a significant increase in S100A8 mRNA expression (Fig. 4**A**). RAGE mRNA (Fig. 4**B**) and protein (Fig. 4**C**) levels were increased likewise. In addition, confocal image analysis of these glial cultures treated with 27-OH (1 μM, 24 h) corroborated these results showing apparent increases in S100A8 and RAGE immunoreactivities, in addition to their co-localization (Fig. 4**D**).

**27-OH-induced RAGE upregulation in astrocytes are mediated by RXRγ**

To decipher the possible mechanism through which 27-OH increases S100A8 and RAGE in astrocytes, we tested the role of the RXRγ, which we previously found mediates detrimental effects of 27-OH on neuron function (36, 37). RXRγ is also expressed in hippocampal astrocytes, has been implicated in regulation of cholesterol metabolism and has been suggested previously as a pharmacological target for AD (38-40). Therefore, we decided to probe RXRγ as a potential mediator of the effects of 27-OH on RAGE expression in astrocytes. RXRγ was knocked-down in astrocytes using siRNA and treatments with 27-OH. As seen by immunohistochemistry in Fig. 5**A**, the effect of high 27-OH on RAGE protein levels in astrocytes was decreased when combined with the RXRγ knockdown. Likewise, RXRγ knockdown blocked the 27-OH induced RAGE increase of protein levels (Fig. 5**B**), as well as mRNA levels (Fig. 5**C**), with a knockdown efficiency of RXRγ of around 80% of control levels (Fig. 5**D**).

**27-OH-induced RAGE increase in neurons is mediated by RXRγ and not by direct LXRβ activation.**

To test if high 27-OH levels were affecting RAGE expression in neurons we used rat primary cortico-hippocampal cultures. When mature neurons were treated with 27-OH 1 µM for 24 hours, we observed again an increase in RAGE protein levels compared to vehicle (Fig. 6**A**). Additionally, treatments with recombinant murine S100A8 12.5 µg/ml for 24h showed significant increased RAGE protein levels (Fig. 6**B**). We previously reported 27-OH induces overexpression of RXRγ in neurons during differentiation, here we treated neurons after 10DIV for 24h with 1 µM of 27-OH and we found no change of RXRγ levels by western blot (Fig. 6**C**, P=0.057). When we knocked down RXRγ using siRNA, we found that RAGE increase with 27-OH was prevented (Fig. 6**D**). When LXRs were blocked using 22(S)-OH treatments, RAGE increase is not prevented in the presence of high 27-OH levels (Fig. S4), which suggest LXRβ activation does not directly induces RAGE expression. Treatments with high 27-OH levels did not induce significant changes on the total levels of NFk-B (Fig. S6**A**) in a similar way as S100A8 did (Fig. S6**B**), nevertheless, NFk-B nuclear translocation was observed in the human neuroblastoma cell line SH-SY5Y (Fig. 6**C**).

**DISCUSSION**

Here we report that excessive 27-OH triggers an alarmin response involving S100A8 and its receptor RAGE in both neurons and astrocytes, *in vivo* and *in vitro*. These results are consistent to the hypothesis proposing that 27-OH mediates detrimental effects of high cholesterol in the plasma over the central nervous system (CNS), as shown by our experiments with HFD mice and in congruence with previous works from our group (15, 16, 33, 37). While more evidence exists of the effect of 27-OH in neurons, our findings propose an astrocytic response to 27-OH involving the S100A8 alarmin.

In the periphery, S100A8 is involved in pathological cascades in multiple diseases such as rheumatoid arthritis (41), systemic erythematosus lupus (42) and cancer (43). In the CNS, S100A8 is related to neurodegenerative diseases such as AD, where the expression of S100A8 is 2-fold higher in the hippocampi of AD patients compared to that of non-demented cases (44). The same is suspected to happen in other neurodegenerative diseases like postoperative cognitive dysfunction (45) and through autophagy alterations in Parkinson´s disease (46). Moreover, treatment with Aβ increased the expression of S100A8 in different paradigms of glial cultures: By 30-fold in microglia chronic treatment after oligomeric Aβ increased the expression of S100A8 (47) and by 2-fold in astrocytes after Aβ_42_ incubation for only 24 h (22). Previously, we also observed that S100A8 treatments increased the levels of Aβ_42_ suggesting a positive feedback between both their productions (22). The intracellular colocalization of RAGE and S100A8 in cultured astrocytes showed in our work suggest a possible response to acute 27-OH treatments through internalization, while we have seen extracellular accumulation of S100A8 aggregates in CYP27Tg brains (data not shown), this could suggest that vesicular traffic would be important in the regulation of S100A8/RAGE signaling and further experiments should be done to explore this avenue.

Our results show that i*n vivo*, both CYP27Tg and the ICV27-OH mouse models, increased their levels of S100A8 and RAGE in the brain as a response to excessive 27-OH levels. RAGE expression in the CNS, promotes neurite outgrowth, neuronal differentiation (25) and participates in repairing injured nerves (48). However, chronic RAGE stimulation affects neuronal function promoting both Tau phosphorylation and Aβ production, which would result in synaptic dysfunction and neurodegeneration (49). Furthermore, the hippocampus of AD patients shows enhanced expressions of RAGE, Aβ and advanced glycation end-products (AGE) as S100A8 (50). Indeed, the increase in RAGE levels found in AD, was shown in neurons, astrocytes, microglia and endothelial cells (51, 52), and it is suggested to contribute to mechanism of AD pathogenesis such us oxidative stress, inflammation, neuronal dysfunction and impairment of short-term memory (53-55).

Data from single cell sequencing shows that S100A8 is not expressed by hippocampal neurons (Figure S3), however it is prominently expressed in the mouse brain (56), so it must be in glial cells mediating the alarmin response such as astrocytes, as seen here by immunocitochemistry. This can be further confirmed consulting AD-oriented databases on single cell expression such as scREAD, which shows microglia and astrocytes overexpressing of S100A8 compared to WT mice in several datasets (57). Given the low expression of neuronal S100A8, the effects of 27-OH over alarmin signaling in neurons is minimal, nevertheless, the induction of S100A8 and RAGE on astrocytes possibly contributes significantly to sterile inflammation. Together with other known neuronal effects (37, 58), high 27-OH levels in the brain can be detrimental for overall brain function due to several cascades that might potentiate between them. In this regard, patients with hereditary spastic paraplegia type 5A (SPG5), have very high 27-OH levels in their brain due to a mutation in the gene CYP7B1 encoding oxysterol-7α-hydroxylase (59). While 27-OH has been found to be neurotoxic, SPG5 patients do not show significant neurodegeneration, which suggest that the interaction between different brain cell types could produce additional metabolites that could play a neuroprotective effect. On the other hand, macrophage activation induces a significant elevation of 25-OH in plasma upon activation (60), but whether this can be translated to microglial activation remains unknown to us. Also, since the CYP27Tg model has a normal CYP7B1 gene, it is possible that some of the effects here described could be mediated by intermediates of 27-OH metabolism like cholestenoic acids or 25-OH, since 27-OH has a rapid catabolism in the brain (61).

We previously found RXRγ is a mediator of 27-OH detrimental effects in neurons (37), however whether RXRγ played a role in astrocytes was unknown. siRNA silencing of RXRγ eliminated the 27-OH-induced increase of RAGE levels in astrocytes as seen by immunohistochemistry, western blot, and qPCR. RXRγ is a nuclear receptor that dimerizes with LXRs and mediates transcription of genes involved in cholesterol and lipid metabolism (62-65). Broad LXR blocking in astrocytes did not prevented RAGE increase mediated by 27-OH (Fig S5). It is possible that 27-OH binds directly to RXRγ or promotes its dimerization with LXR or another nuclear receptor, which have been shown to bind oxysterols (66). We showed that a similar mechanism regulating RAGE expression is present in neurons, since knockdown of RXRγ prevented 27-OH induced RAGE expression at the mRNA level. Also, 27-OH induction of RAGE in primary neurons was independent of the presence of S100A8, meaning that high levels of 27-OH can sensitize neurons to alarmin signaling by increasing the amount of RAGE receptors before inflammation takes place (Fig 7). This becomes evident when treating a human neuroblastoma cell line with high levels of 27-OH, which induces nuclear translocation of NFk-B (Fig. S5). Finally, we propose the effect of 27-OH over neuronal alarmin cascades can take place in humans since similar effects are found on human neuroblastoma cells (Fig. S1 and Fig. S5).

**CONCLUSIONS.**

Our data supports the notion that high levels of peripheral cholesterol generate in turn increased 27-OH levels and enhance an inflammatory signaling cascade in the brain. HFD and excessive 27-OH result in S100A8 accumulation, increased RAGE expression, and therefore enhances alarmin cascades mediated by RXRγ (20, 22). These results places 27-OH as a mediator of the pathological mechanisms linking hypercholesterolemia and sterile inflammation in the brain, with potential implication in the pathophysiology of AD and other neurodegenerative diseases.

**List of abbreviations.**

Alzheimer´s disease (AD)

Apolipoprotein E, allele epsilon 4 (APOEε4)

Clusterin (ApoJ)

Blood-brain barrier (BBB)

27-hydroxycholesterol (27-OH)

CYP27A1 overexpressor (CYP27Tg)

Cerebrospinal fluid (CSF)

Receptor for Advanced Glycation End product (RAGE)

Retinoid X Receptor gamma (RXRγ)

High fat/high cholesterol diet (HFD)

Wild-type mice (WT) fed with HFD (WT-HFD)

Wild-type mice (WT) fed with normal diet (WT-ND)

Corpora amylacea (CA)

Intracerebroventricular injections (ICV)

artificial cerebrospinal fluid (aCSF)

Central nervous system (CNS)

High-density lipoprotein (HDL)

Nuclear factor kappa-light-chain-enhancer of activated B cells (NFkB).

Hereditary spastic paraplegia type 5A (SPG5).

**DECLARATIONS.**

*Funding.* This research was supported by the following Swedish foundations: Swedish Brain Power, the regional agreement on medical training and clinical research (ALF) between Stockholm County Council and Karolinska Institutet, Strategic Neuroscience Program, Margaretha af Ugglas Foundation, Gun och Bertil Stohnes Stiftelse, Karolinska Institutet fund for geriatric research, Stiftelsen Gamla Tjänarinnor, Demensfonden, Lindhés Advokatbyrå, Hjärnfonden and Alzheimerfonden. R.L-V was financially supported by Mexico’s National Council for Science and Technology (CONACYT) CVU: 209252, and by Olle Enqvist Foundation grant no. 2014/778. Ramon Areces Foundation, Spain, supported E.P., EMBO Long-Term Fellowship (ALTF 696-2013), the SSMF postdoctoral Fellowship and Juan de la Cierva-Incorporación.

(IJCI-2016-27658) supported P. M-S.

*Competing interests*. The authors declare no conflict of interest with this work.

*Data Availability*. Data for this work is archived and publicly available upon request to the corresponding authors, as well as at the Karolinska Institutet repository and the Swedish National Archive.

*Code availability.* Software used in this work is commercially available. No custom coding was done for this work.

*Author contributions.* Conceptualization, A.C-M., I.B. and E.P.; Methodology, J.G., A.C.-M., E.P., R.L-V., M.L. and M.-A.-M.I.; Investigation, M.-A.-M.I., M.L., E.P., R.L-V., L.M., P.R-R., S.M. and P.M.-S.; S100A8 Protein production and purification, C.G., M. A.R.; Formal Analysis, M.-A.-M.I., M.L., E.P., R.L-V. and L.M.; Writing – Original Draft, M.-A.-M.I., R.L-V., M.L., and A.C.-M.; Writing – Review & Editing, All authors; Funding Acquisition, A.C.-M., M.-A.-AM.I., R.L-V., I.B., M.L., S.M. and E.P. All authors made substantial contributions to the manuscript. All authors read and approved the submitted manuscript.

*Ethics approval.* All experimental procedures with animals were conducted following European relevant guidelines and regulations and were approved by the Swedish Board of Agriculture (ethical permits ID S33-13, extension 57-15 and 4884/2019).

*Consent to participate.* Not applicable. This work does not include human patient’s samples.

*Consent for publication.* Not applicable. This work does not have human patient’s samples.

*Acknowledgements*. The imaging for this study was performed at the Live Cell Imaging Facility, Karolinska Institutet, Sweden, supported by grants from the Knut and Alice Wallenberg Foundation, the Swedish Research Council, the Centre for Innovative Medicine and the Jonasson Center at the Royal Institute of Technology, Sweden.

**REFERENCES**

1. Anstey KJ, Ashby-Mitchell K, Peters R. Updating the Evidence on the Association between Serum Cholesterol and Risk of Late-Life Dementia: Review and Meta-Analysis. J Alzheimers Dis. 2017;56(1):215-28.

2. Anstey KJ, Lipnicki DM, Low LF. Cholesterol as a risk factor for dementia and cognitive decline: a systematic review of prospective studies with meta-analysis. The American journal of geriatric psychiatry : official journal of the American Association for Geriatric Psychiatry. 2008;16(5):343-54.

3. Kivipelto M, Rovio S, Ngandu T, Kareholt I, Eskelinen M, Winblad B, et al. Apolipoprotein E epsilon4 magnifies lifestyle risks for dementia: a population-based study. Journal of cellular and molecular medicine. 2008;12(6B):2762-71.

4. Solomon A, Kareholt I, Ngandu T, Winblad B, Nissinen A, Tuomilehto J, et al. Serum cholesterol changes after midlife and late-life cognition: twenty-one-year follow-up study. Neurology. 2007;68(10):751-6.

5. Kivipelto M. Midlife vascular risk factors and Alzheimer's disease in later life: longitudinal, population based study. BMJ. 2001;322(7300):1447-51.

6. Corder E, Saunders A, Strittmatter W, Schmechel D, Gaskell P, Small G, et al. Gene dose of apolipoprotein E type 4 allele and the risk of Alzheimer's disease in late onset families. Science. 1993;261(5123):921-3.

7. Harold D, Abraham R, Hollingworth P, Sims R, Gerrish A, Hamshere ML, et al. Genome-wide association study identifies variants at CLU and PICALM associated with Alzheimer's disease. Nat Genet. 2009;41(10):1088-93.

8. Hollingworth P, Harold D, Sims R, Gerrish A, Lambert J-C, Carrasquillo MM, et al. Common variants in ABCA7, MS4A6A/MS4A4E, EPHA1, CD33 and CD2AP are associated with Alzheimer’s disease. Nature genetics. 2011;43(5):429-35.

9. Kivipelto M, Helkala E-L, Laakso MP, Hänninen T, Hallikainen M, Alhainen K, et al. Midlife vascular risk factors and Alzheimer's disease in later life: longitudinal, population based study. BMJ. 2001;322(7300):1447-51.

10. Shepardson NE, Shankar GM, Selkoe DJ. Cholesterol level and statin use in Alzheimer disease: II. Review of human trials and recommendations. Arch Neurol. 2011;68(11):1385-92.

11. Reitz C, Tang MX, Manly J, Schupf N, Mayeux R, Luchsinger JA. Plasma lipid levels in the elderly are not associated with the risk of mild cognitive impairment. Dement Geriatr Cogn Disord. 2008;25(3):232-7.

12. Puglielli L, Tanzi RE, Kovacs DM. Alzheimer's disease: the cholesterol connection. Nat Neurosci. 2003;6(4):345-51.

13. Bjorkhem I, Cedazo-Minguez A, Leoni V, Meaney S. Oxysterols and neurodegenerative diseases. Molecular aspects of medicine. 2009;30(3):171-9.

14. Mateos L, Akterin S, Gil-Bea FJ, Spulber S, Rahman A, Bjorkhem I, et al. Activity-regulated cytoskeleton-associated protein in rodent brain is down-regulated by high fat diet in vivo and by 27-hydroxycholesterol in vitro. Brain pathology. 2009;19(1):69-80.

15. Ismail MA, Mateos L, Maioli S, Merino-Serrais P, Ali Z, Lodeiro M, et al. 27-Hydroxycholesterol impairs neuronal glucose uptake through an IRAP/GLUT4 system dysregulation. J Exp Med. 2017;214(3):699-717.

16. Heverin M, Maioli S, Pham T, Mateos L, Camporesi E, Ali Z, et al. 27-hydroxycholesterol mediates negative effects of dietary cholesterol on cognition in mice. Behavioural brain research. 2015;278:356-9.

17. Umetani M, Ghosh P, Ishikawa T, Umetani J, Ahmed M, Mineo C, et al. The Cholesterol Metabolite 27-Hydroxycholesterol Promotes Atherosclerosis via Proinflammatory Processes Mediated by Estrogen Receptor Alpha. Cell Metabolism. 2014;20(1):172-82.

18. Heverin M, Bogdanovic N, Lutjohann D, Bayer T, Pikuleva I, Bretillon L, et al. Changes in the levels of cerebral and extracerebral sterols in the brain of patients with Alzheimer's disease. Journal of lipid research. 2004;45(1):186-93.

19. Calsolaro V, Edison P. Neuroinflammation in Alzheimer's disease: Current evidence and future directions. Alzheimer's & Dementia. 2016;12(6):719-32.

20. Vogl T, Stratis A, Wixler V, Voller T, Thurainayagam S, Jorch SK, et al. Autoinhibitory regulation of S100A8/S100A9 alarmin activity locally restricts sterile inflammation. J Clin Invest. 2018;128(5):1852-66.

21. Shepherd CE, Goyette J, Utter V, Rahimi F, Yang Z, Geczy CL, et al. Inflammatory S100A9 and S100A12 proteins in Alzheimer's disease. Neurobiol Aging. 2006;27(11):1554-63.

22. Lodeiro M, Puerta E, Ismail M-A-M, Rodriguez-Rodriguez P, Rönnbäck A, Codita A, et al. Aggregation of the Inflammatory S100A8 Precedes Aβ Plaque Formation in Transgenic APP Mice: Positive Feedback for S100A8 and Aβ Productions. The Journals of Gerontology Series A: Biological Sciences and Medical Sciences. 2016.

23. Xie J, Méndez JD, Méndez-Valenzuela V, Aguilar-Hernández MM. Cellular signalling of the receptor for advanced glycation end products (RAGE). Cellular Signalling. 2013;25(11):2185-97.

24. Park IH, Yeon SI, Youn JH, Choi JE, Sasaki N, Choi IH, et al. Expression of a novel secreted splice variant of the receptor for advanced glycation end products (RAGE) in human brain astrocytes and peripheral blood mononuclear cells. Molecular immunology. 2004;40(16):1203-11.

25. Huttunen HJ, Kuja-Panula J, Sorci G, Agneletti AL, Donato R, Rauvala H. Coregulation of neurite outgrowth and cell survival by amphoterin and S100 proteins through receptor for advanced glycation end products (RAGE) activation. The Journal of biological chemistry. 2000;275(51):40096-105.

26. Yan SF, Yan SD, Ramasamy R, Schmidt AM. Tempering the wrath of RAGE: an emerging therapeutic strategy against diabetic complications, neurodegeneration, and inflammation. Annals of medicine. 2009;41(6):408-22.

27. Meir K, Kitsberg D, Alkalay I, Szafer F, Rosen H, Shpitzen S, et al. Human sterol 27-hydroxylase (CYP27) overexpressor transgenic mouse model. Evidence against 27-hydroxycholesterol as a critical regulator of cholesterol homeostasis. The Journal of biological chemistry. 2002;277(37):34036-41.

28. Cedazo-Mı́nguez A, Hamker U, Meske V, Veh RW, Hellweg R, Jacobi C, et al. Regulation of apolipoprotein E secretion in rat primary hippocampal astrocyte cultures. Neuroscience. 2001;105(3):651-61.

29. Brophy MB, Hayden JA, Nolan EM. Calcium Ion Gradients Modulate the Zinc Affinity and Antibacterial Activity of Human Calprotectin. Journal of the American Chemical Society. 2012;134(43):18089-100.

30. Hagmeyer S, Romão MA, Cristóvão JS, Vilella A, Zoli M, Gomes CM, et al. Distribution and Relative Abundance of S100 Proteins in the Brain of the APP23 Alzheimer’s Disease Model Mice. Frontiers in Neuroscience. 2019;13(640).

31. Botelho HM, Fritz G, Gomes CM. Analysis of S100 Oligomers and Amyloids. In: Sigurdsson EM, Calero M, Gasset M, editors. Amyloid Proteins: Methods and Protocols. Methods in Molecular Biology. New York: Springer Science+Business Media; 2012.

32. Mateos L, Ismail MA, Gil-Bea FJ, Schule R, Schols L, Heverin M, et al. Side chain-oxidized oxysterols regulate the brain renin-angiotensin system through a liver X receptor-dependent mechanism. The Journal of biological chemistry. 2011;286(29):25574-85.

33. Maioli S, Puerta E, Merino-Serrais P, Fusari L, Gil-Bea F, Rimondini R, et al. Combination of apolipoprotein E4 and high carbohydrate diet reduces hippocampal BDNF and arc levels and impairs memory in young mice. Journal of Alzheimer's disease : JAD. 2012;32(2):341-55.

34. Hoyaux D, Decaestecker C, Heizmann CW, Vogl T, Schafer BW, Salmon I, et al. S100 proteins in Corpora amylacea from normal human brain. Brain Res. 2000;867(1-2):280-8.

35. Heverin M, Maioli S, Pham T, Mateos L, Camporesi E, Ali Z, et al. 27-Hydroxycholesterol mediates negative effects of dietary cholesterol on cognition in mice. Behavioural brain research. 2015;278:356-9.

36. Baranowski M. Biological role of liver X receptors. J Physiol Pharmacol. 2008;59(Suppl 7):31-55.

37. Merino-Serrais P, Loera-Valencia R, Rodriguez-Rodriguez P, Parrado-Fernandez C, Ismail MA, Maioli S, et al. 27-Hydroxycholesterol Induces Aberrant Morphology and Synaptic Dysfunction in Hippocampal Neurons. Cereb Cortex. 2019;29(1):429-46.

38. Repa JJ, Turley SD, Lobaccaro JA, Medina J, Li L, Lustig K, et al. Regulation of absorption and ABC1-mediated efflux of cholesterol by RXR heterodimers. Science. 2000;289(5484):1524-9.

39. Dheer Y, Chitranshi N, Gupta V, Abbasi M, Mirzaei M, You Y, et al. Bexarotene Modulates Retinoid-X-Receptor Expression and Is Protective Against Neurotoxic Endoplasmic Reticulum Stress Response and Apoptotic Pathway Activation. Molecular neurobiology. 2018;55(12):9043-56.

40. Zeisel A, Munoz-Manchado AB, Codeluppi S, Lonnerberg P, La Manno G, Jureus A, et al. Brain structure. Cell types in the mouse cortex and hippocampus revealed by single-cell RNA-seq. Science. 2015;347(6226):1138-42.

41. Sunahori K, Yamamura M, Yamana J, Takasugi K, Kawashima M, Yamamoto H, et al. The S100A8/A9 heterodimer amplifies proinflammatory cytokine production by macrophages via activation of nuclear factor kappa B and p38 mitogen-activated protein kinase in rheumatoid arthritis. Arthritis Res Ther. 2006;8(3):R69.

42. Soyfoo MS, Roth J, Vogl T, Pochet R, Decaux G. Phagocyte-specific S100A8/A9 protein levels during disease exacerbations and infections in systemic lupus erythematosus. J Rheumatol. 2009;36(10):2190-4.

43. Ghavami S, Rashedi I, Dattilo BM, Eshraghi M, Chazin WJ, Hashemi M, et al. S100A8/A9 at low concentration promotes tumor cell growth via RAGE ligation and MAP kinase-dependent pathway. J Leukoc Biol. 2008;83(6):1484-92.

44. Lue LF, Kuo YM, Beach T, Walker DG. Microglia activation and anti-inflammatory regulation in Alzheimer's disease. Molecular neurobiology. 2010;41(2-3):115-28.

45. Lu SM, Yu CJ, Liu YH, Dong HQ, Zhang X, Zhang SS, et al. S100A8 contributes to postoperative cognitive dysfunction in mice undergoing tibial fracture surgery by activating the TLR4/MyD88 pathway. Brain Behav Immun. 2015;44:221-34.

46. Ghavami S, Eshragi M, Ande SR, Chazin WJ, Klonisch T, Halayko AJ, et al. S100A8/A9 induces autophagy and apoptosis via ROS-mediated cross-talk between mitochondria and lysosomes that involves BNIP3. Cell Res. 2010;20(3):314-31.

47. Walker DG, Link J, Lue LF, Dalsing-Hernandez JE, Boyes BE. Gene expression changes by amyloid beta peptide-stimulated human postmortem brain microglia identify activation of multiple inflammatory processes. J Leukoc Biol. 2006;79(3):596-610.

48. Rong LL, Yan SF, Wendt T, Hans D, Pachydaki S, Bucciarelli LG, et al. RAGE modulates peripheral nerve regeneration via recruitment of both inflammatory and axonal outgrowth pathways. FASEB J. 2004;18(15):1818-25.

49. Li XH, Lv BL, Xie JZ, Liu J, Zhou XW, Wang JZ. AGEs induce Alzheimer-like tau pathology and memory deficit via RAGE-mediated GSK-3 activation. Neurobiol Aging. 2012;33(7):1400-10.

50. Valente T, Gella A, Fernandez-Busquets X, Unzeta M, Durany N. Immunohistochemical analysis of human brain suggests pathological synergism of Alzheimer's disease and diabetes mellitus. Neurobiol Dis. 2010;37(1):67-76.

51. Sasaki N, Toki S, Chowei H, Saito T, Nakano N, Hayashi Y, et al. Immunohistochemical distribution of the receptor for advanced glycation end products in neurons and astrocytes in Alzheimer's disease. Brain Res. 2001;888(2):256-62.

52. Miller MC, Tavares R, Johanson CE, Hovanesian V, Donahue JE, Gonzalez L, et al. Hippocampal RAGE immunoreactivity in early and advanced Alzheimer's disease. Brain Res. 2008;1230:273-80.

53. Fang F, Lue LF, Yan S, Xu H, Luddy JS, Chen D, et al. RAGE-dependent signaling in microglia contributes to neuroinflammation, Abeta accumulation, and impaired learning/memory in a mouse model of Alzheimer's disease. FASEB J. 2010;24(4):1043-55.

54. Arancio O, Zhang HP, Chen X, Lin C, Trinchese F, Puzzo D, et al. RAGE potentiates Abeta-induced perturbation of neuronal function in transgenic mice. The EMBO journal. 2004;23(20):4096-105.

55. Origlia N, Righi M, Capsoni S, Cattaneo A, Fang F, Stern DM, et al. Receptor for advanced glycation end product-dependent activation of p38 mitogen-activated protein kinase contributes to amyloid-beta-mediated cortical synaptic dysfunction. J Neurosci. 2008;28(13):3521-30.

56. Hagmeyer S, Romao MA, Cristovao JS, Vilella A, Zoli M, Gomes CM, et al. Distribution and Relative Abundance of S100 Proteins in the Brain of the APP23 Alzheimer's Disease Model Mice. Front Neurosci. 2019;13:640.

57. Jiang J, Wang C, Qi R, Fu H, Ma Q. scREAD: A Single-Cell RNA-Seq Database for Alzheimer's Disease. iScience. 2020;23(11):101769.

58. Loera-Valencia R, Vazquez-Juarez E, Munoz A, Gerenu G, Gomez-Galan M, Lindskog M, et al. High levels of 27-hydroxycholesterol results in synaptic plasticity alterations in the hippocampus. Sci Rep. 2021;11(1):3736.

59. Schols L, Rattay TW, Martus P, Meisner C, Baets J, Fischer I, et al. Hereditary spastic paraplegia type 5: natural history, biomarkers and a randomized controlled trial. Brain. 2017;140(12):3112-27.

60. Diczfalusy U. On the formation and possible biological role of 25-hydroxycholesterol. Biochimie. 2013;95(3):455-60.

61. Meaney S, Heverin M, Panzenboeck U, Ekström L, Axelsson M, Andersson U, et al. Novel route for elimination of brain oxysterols across the blood-brain barrier: conversion into 7α-hydroxy-3-oxo-4-cholestenoic acid. Journal of lipid research. 2007;48(4):944-51.

62. Wang H, Chu W, Hemphill C, Hasstedt SJ, Elbein SC. Mutation screening and association of human retinoid X receptor gamma variation with lipid levels in familial type 2 diabetes. Mol Genet Metab. 2002;76(1):14-22.

63. Nohara A, Kawashiri MA, Claudel T, Mizuno M, Tsuchida M, Takata M, et al. High frequency of a retinoid X receptor gamma gene variant in familial combined hyperlipidemia that associates with atherogenic dyslipidemia. Arterioscler Thromb Vasc Biol. 2007;27(4):923-8.

64. Zhao C, Dahlman-Wright K. Liver X receptor in cholesterol metabolism. J Endocrinol. 2010;204(3):233-40.

65. Fan H, Dong W, Li Q, Zou X, Zhang Y, Wang J, et al. Ajuba Preferentially Binds LXRalpha/RXRgamma Heterodimer to Enhance LXR Target Gene Expression in Liver Cells. Mol Endocrinol. 2015;29(11):1608-18.

66. Wada T, Kang HS, Angers M, Gong H, Bhatia S, Khadem S, et al. Identification of oxysterol 7alpha-hydroxylase (Cyp7b1) as a novel retinoid-related orphan receptor alpha (RORalpha) (NR1F1) target gene and a functional cross-talk between RORalpha and liver X receptor (NR1H3). Mol Pharmacol. 2008;73(3):891-9.

**FIGURES**

**
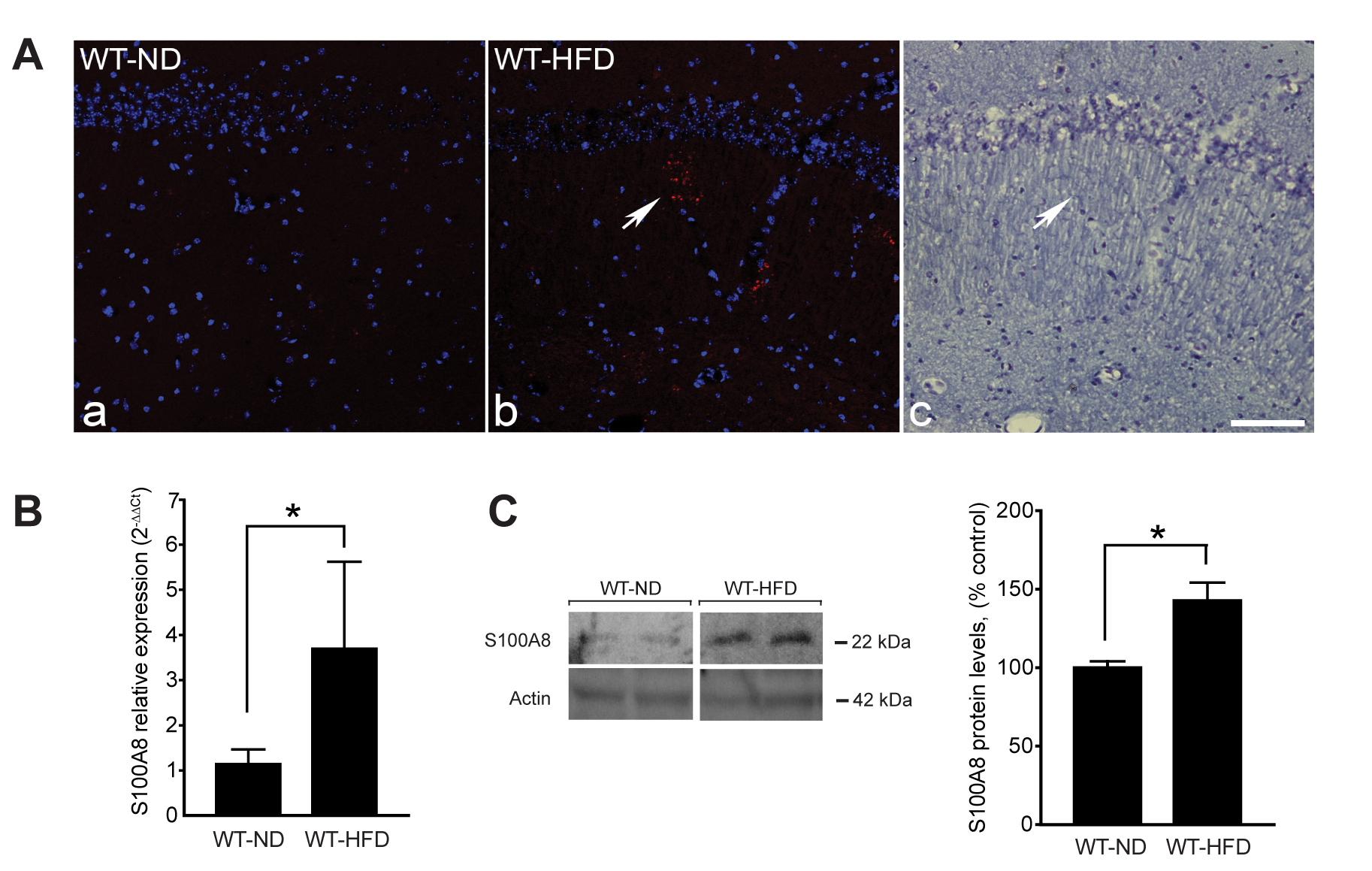
**

**Figure 1.** **High fat diet (HFD) and 27-OH enhances the expression of S100A8 *in vivo.*** (**A**) Confocal microscopy of sections double stained with anti-S100A8 (red) and DAPI (blue) from the same field in the CA1 hippocampal region of WT mice on a normal diet (WT-ND) (*a*) or a high fat diet (WT-HFD) (*b*). In (*c*) the same section as (*b*) stained with cresyl violet showing, with an arrow, the S100A8 aggregates. Scale bar in (c): 120µm. (**B**) S100A8 mRNA expression levels in the hippocampus of WT-ND (Mean=1.34, SEM=0.37, n=8 animals) and WT-HFD (Mean=4.038, SEM=1.22, n=7 animals, P=0.04). (**C**) A representative western blot showing protein levels of S100A8 of ND mice (Mean=100.0, SEM=4.077, n=4 animals) and HFD mice (Mean=142.8, SEM=11.42, n=4 animals, P=0.012).


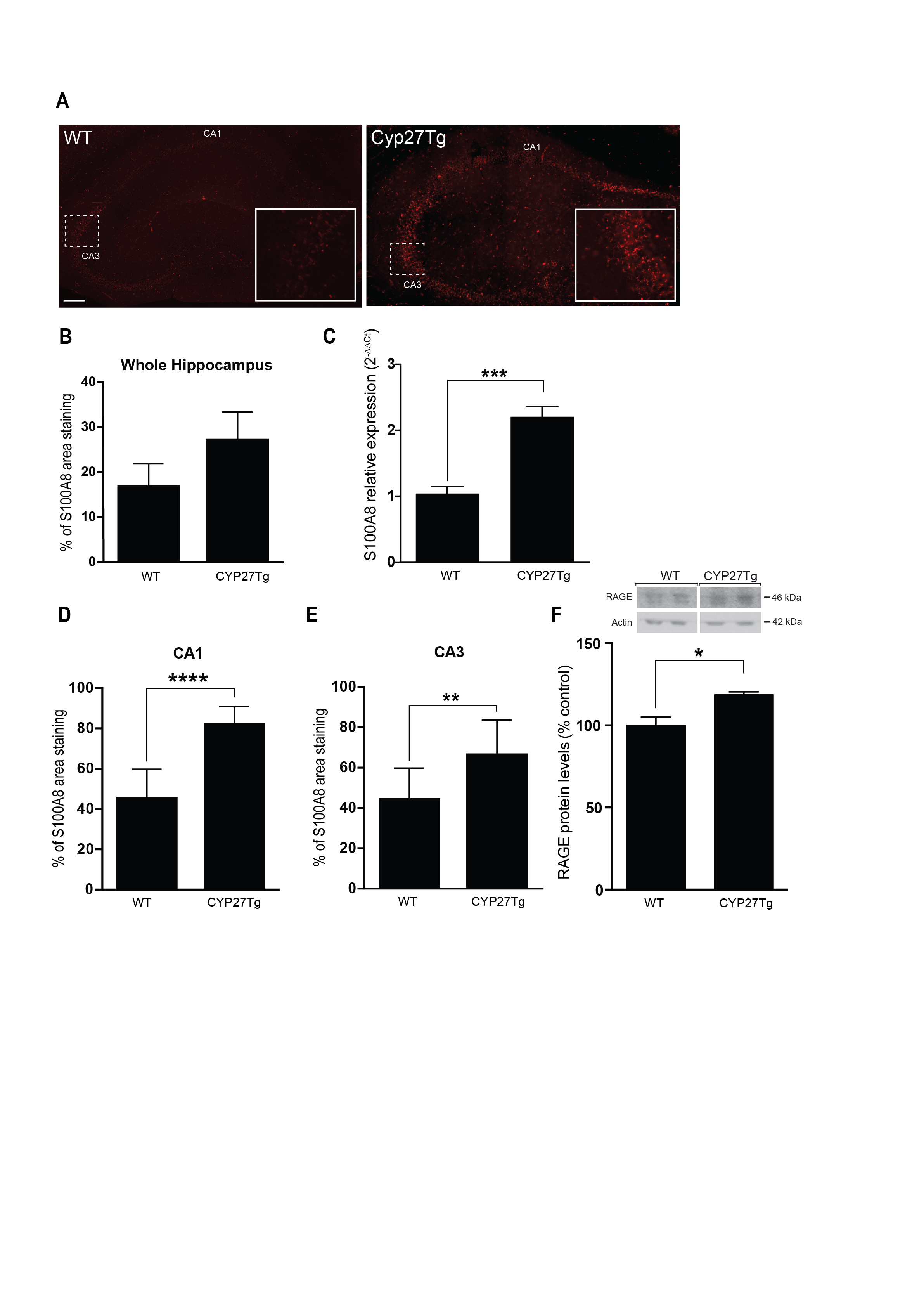


**Figure 2. 27-OH increases S100A8 and RAGE in CYP27Tg mice. A)** S100A8 immunofluorescence in the hippocampus of CYP27Tg mouse model, overexpressing the human *CYP27A1* gene, versus wild type mice (12 m.o.) by confocal microscopy. Inserts (i and ii) shows a region in CA3 with S100A8 staining in WT and CYP27Tg respectively. Bar = 300µm. **B)** Quantification of S100A8 area immuno-stained in whole hippocampi of CYP27Tg mice (WTmean=16.67, SEM=3.038; CYP27Tgmean=27.12, SEM=3.097 n=4 animals per group, P=0.06). **C)** Levels of S100A8 mRNA in CYP27Tg hippocampi vs. WT counterparts (12 m.o. p < 0.0001, n=4 animals per group). **D)** Quantification of S100A8 area immuno-stained in CA1 regions of CYP27Tg mice (Mean 81.83, SEM= 2.84, p < 0.0001, n=10 fields from 4 animals). **E)** Quantification of S100A8 area immuno-stained in CA3 region of hippocampus of WT (Mean=44.16, SEM=5.16, n=9 fields from 4 animals) vs CYP27Tg (Mean=66.32, SEM=5.46, n=10 fields from 4 animals, P=0.0094). **F)** Protein levels of RAGE in whole lysates from CYP27Tg mice (Mean=117.9, SEM=1,64, n=3 animals) vs. WT mice at 12 m.o. (Mean=100, SEM=5,57, n=4 animals, P=0.044).


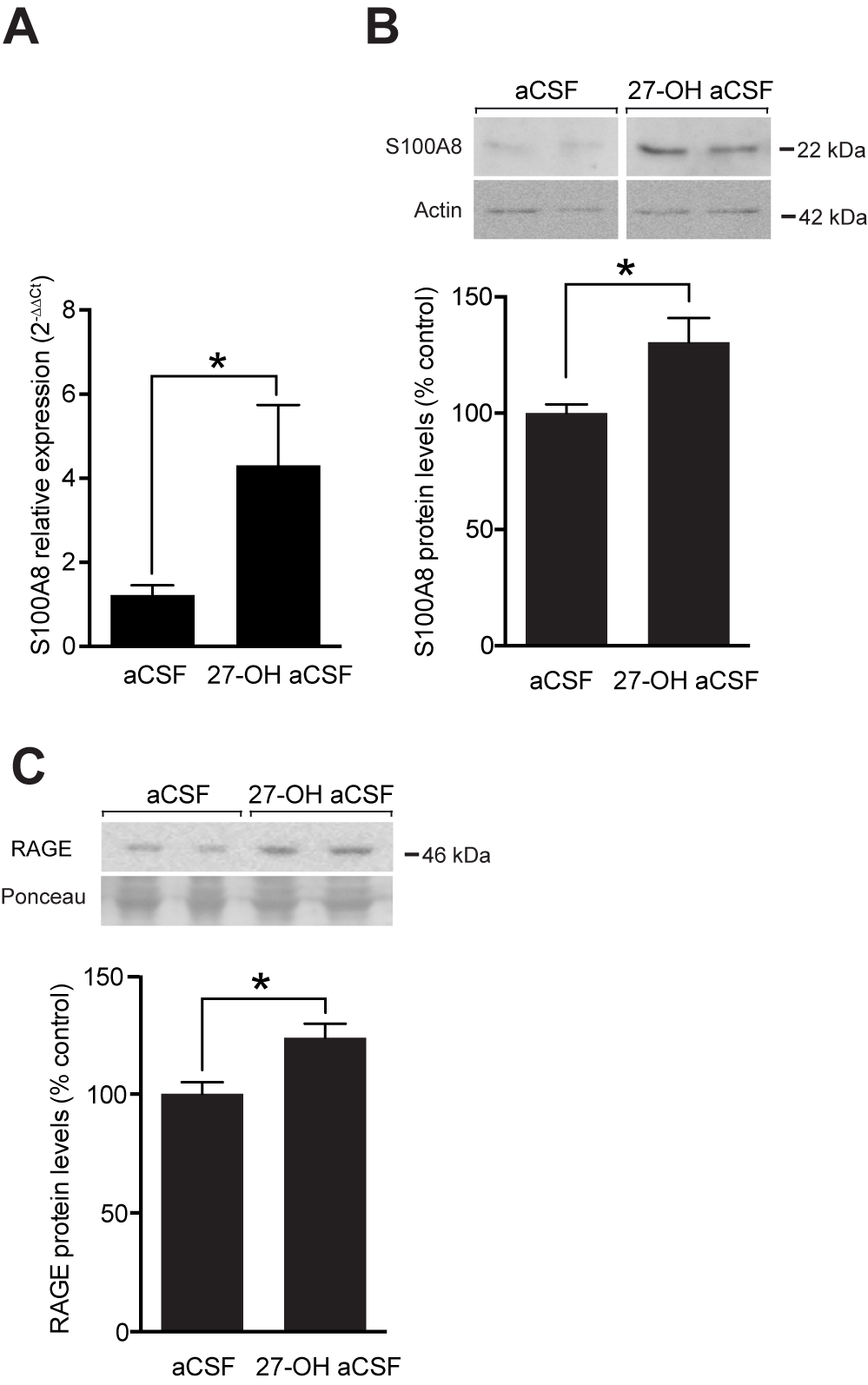


**Fig. 3.** **27-OH increases S100A8 and RAGE expressions administered acutely in the brain.** Intracerebroventricularly injected 27-OH (ICV27-OH) (10 μM) into the lateral ventricle of wild-type mice showing (**A**) mRNA levels of S100A8 in aCSF controls (Mean=1.22, SEM=0.23, n=4 animals, P=0.018) and on 27-OH mice (Mean=4.30, SEM=1.43, n=4 animals). Protein levels by western blot of S100A8 (**B**, WTmean=100, SEM=3.81, n=4 animals; CYP27Tgmean=110.3, SEM=10.3, n=4 animals, P=0.025) and RAGE (**C,** WTmean=100, SEM=34.97, n=4 animals; CYP27Tgmean=123.8, SEM=5.94, n= 4 animals, P=0.021) of ICV27-OH.

**
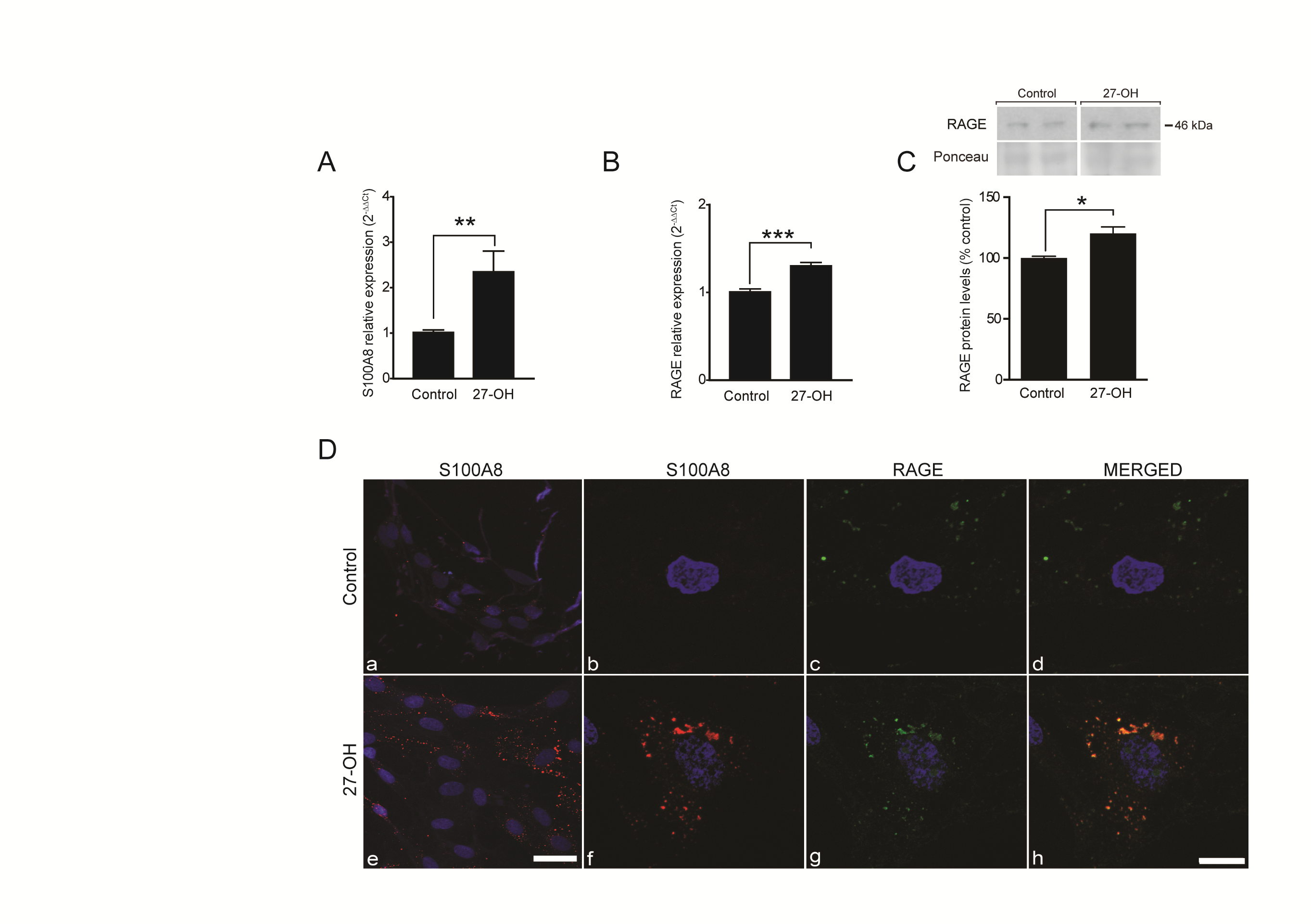
**

**Figure 4. 27-OH increases S100A8 and RAGE expressions in astrocytes*.* A**) Expression levels of S100A8 in glial cells from primary rat cultures treated with vehicle (CNTmean=1.016, SEM=.05, n=12 wells) or with 27-OH (1 μM, 24 h, 27-OHmean=2.350, SEM=0.45, n=12 wells, P=0.008) by RT-qPCR. **B**) RAGE mRNA levels in glial cells from primary rat cultures treated with vehicle (CNTmean=1.007, SEM=0.13, n=15 wells) or with 27-OH (1 μM, 24 h, 27-OHmean=1.304, SEM=0.037, n=16 wells, P< 0.0001). **C**) Confocal microscopy of glial cell cultures untreated (upper panel) or treated (lower panel) with 27-OH (1 μM, 24 h) and stained with anti-S100A8 (red), anti- RAGE (green) and DAPI (blue). (a) and (e) are overview images. Scale bar in (e): 75 µm. For all other panels refer to scale in (*h*): 25 µm.

**
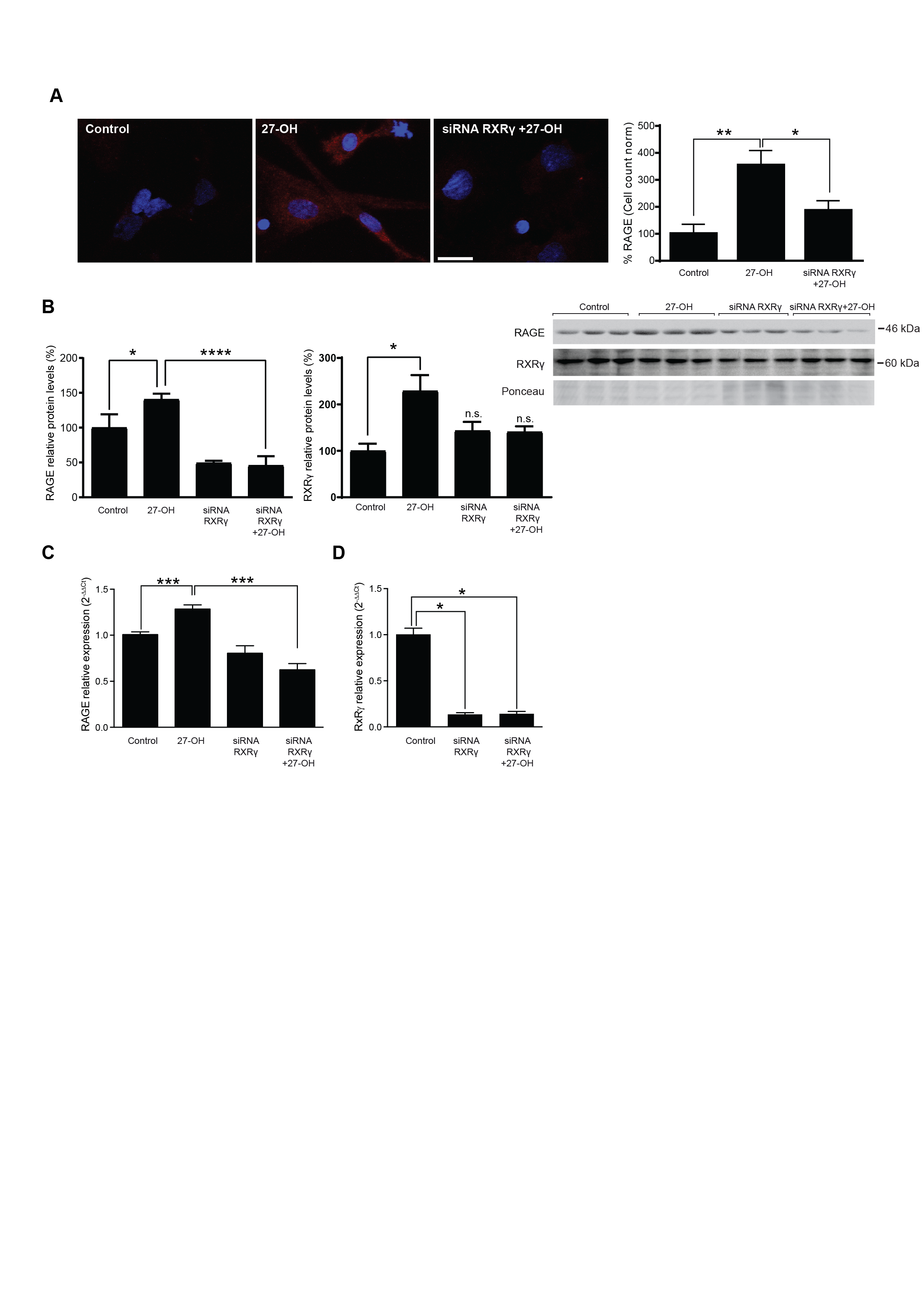
Figure 5. 27-OH induction of RAGE in astrocytes is mediated by RXRγ.** (**A**) Knockdown of RXRγ was done using siRNA. RAGE protein levels were increased in astrocytes in culture with 27-OH treatment (1µM) and prevented with knocking down of RXRγ as observed by immunostaining acquired by confocal imaging (% of area staining normalized to cell number; 1 field per well cultured. Controlmean=100, SEM=20.4, n=3 fields; 27-OHmean=353.8, SEM=55.32, n=4 fields; iRXRg+27-OHmean=185.9, SEM=18.34, n=4 fields. ANOVA P=0.005. Tukey´s multiple comparisons (*) P<0.05, (**) P<0.001). (**B**) Knock-down of RXRγ prevented the increase of RAGE mediated by 27-OH (1µM) at the protein level (Controlmean=100, SEM=11.01, n=3 wells; 27-OHmean=140.4, SEM=4.815, n=3 wells; iRXRgmean=49.22, SEM=1.824, n=3wells; iRxRg+27-OHmean=45.62, SEM=7.70, n=3wells. ANOVA P<0.0001. Tukey´s multiple comparisons (*) P=0.0173, (****) P ≤.0001). RXRγ densitometry shows its increase by 27-OH treatment, which is prevented by siRNA (Controlmean=100, SEM=15.37, n=3 wells; 27-OHmean=228.4, SEM=34.37, n=3 wells; iRXRgmean=143.1, SEM=19.27, n=3wells; iRXRg+27-OHmean=140.7, SEM=12.09, n=3wells. ANOVA P=0.028. Tukey´s multiple comparisons (*) P=0.02; n.s. iRXRg = 0.27; n.s. iRXRg+27-OH=0.68). (**C**) mRNA levels of RAGE were diminished in the RXRγ knocked-down astrocytes treated with 27-OH (Controlmean=1.007, SEM=0.03, n=18 wells; 27-OHmean=1.284, SEM=0.04, n=19 wells; iRXRgmean=0.804, SEM=0.08, n=6 wells; iRXRg+27-OHmean=0.6241, SEM=0.06, n=6 wells. ANOVA P<0.0001. Tukey´s multiple comparisons (***) P ≤.001). (**D**) RXRγ knock-down prevented its own upregulation by 27-OH (Controlmean=1.004, SEM=0.06, n=3 wells; iRXRgmean=0.1354, SEM=0.019, n=3 wells; iRXRg+27-OHmean=0.14, SEM=0.02, n=3 wells. ANOVA P<0.0001. Tukey´s multiple comparisons (*) P<0.05).


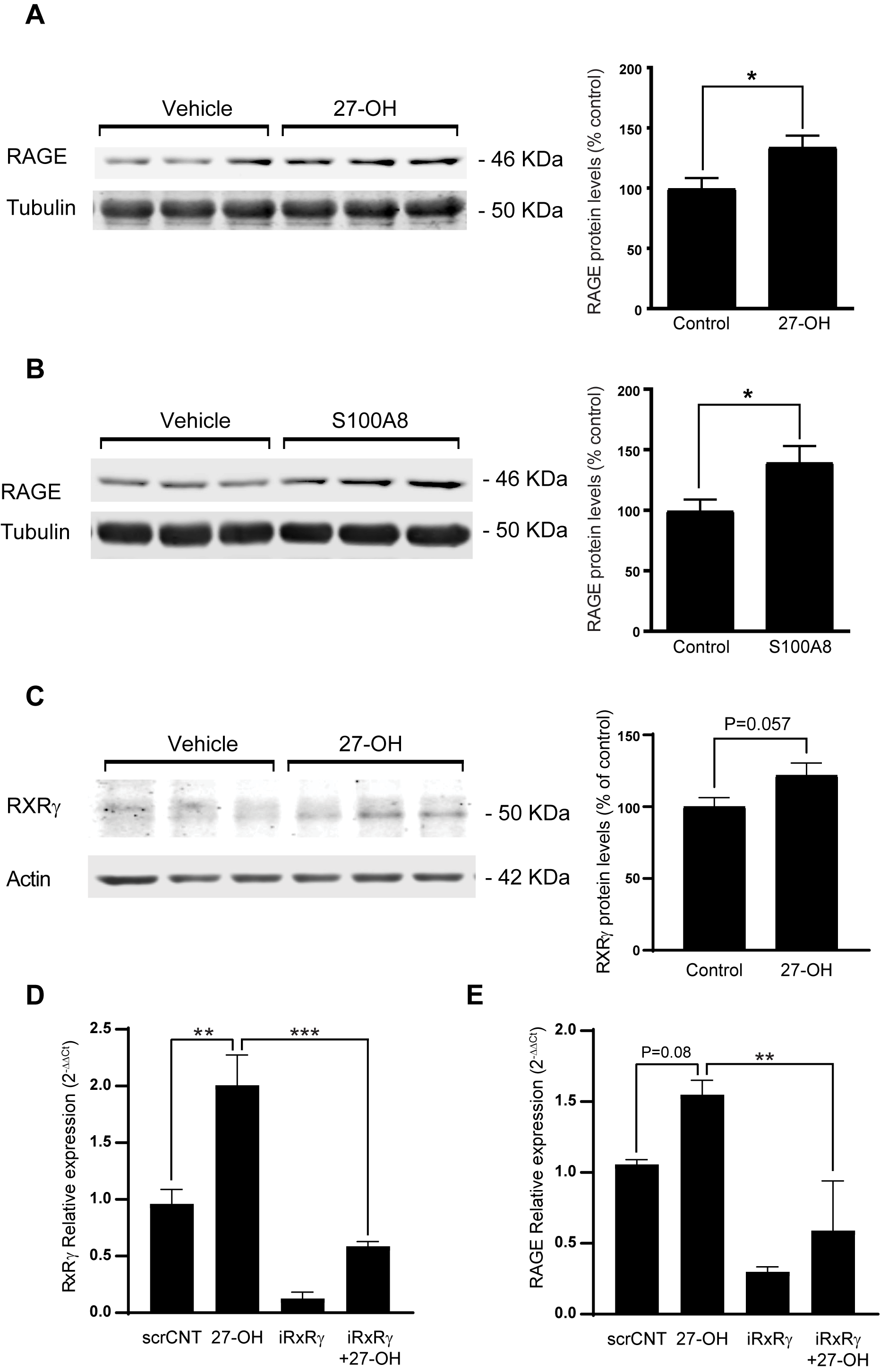


**Figure 6. 27-OH-induced RAGE increase in neurons.** (**A**) Western blots from rat primary neurons treated with 27-OH (1 μM, 24 h) showing protein levels of RAGE (Vehiclemean=1.0, SEM=0.08, n=8 wells; 27-OHmean=1.34, SEM=0.09, n=8 wells. Unpaired t test P=0.01) (**B**) Western blots from rat primary cultures treated with S100A8 (12.5 ug/ml, 24h) showing protein levels of RAGE (Vehiclemean=1.0, SEM=0.08, n=12 wells; 27-OHmean=1.39, SEM=0.13, n=12 wells. Unpaired t test P=0.02) (**C**) Western blots from rat primary neurons treated with 27-OH (1 μM, 24 h) showing protein levels of RXRγ (Vehiclemean=1.0, SEM=0.08, n=8 wells; 27-OHmean=1.34, SEM=0.09, n=8 wells. Unpaired t test P=0.053) and (**D**) mRNA levels of RXRγ were diminished in the RXRγ-knocked-down primary neurons treated with 27-OH (Controlmean=0.96, SEM=0.1247, n=4 wells; 27-OHmean=2.007, SEM=0.26, n=6 wells; iRXRgmean=0.12, SEM=0.056, n=4 wells; iRXRg+27-OHmean=0.58, SEM=0.04, n=4 wells. ANOVA P<0.0001. Tukey´s multiple comparisons (**) P=0.005; (***) P=0.002) and a similar effect was found on RAGE mRNA expression (Controlmean=1.057, SEM=0.033, n=4 wells; 27-OHmean=1.54, SEM=0.101, n=4 wells; iRXRgmean=0.29, SEM=0.035, n=4 wells; iRXRg+27-OHmean=0.59, SEM=0.35, n=4 wells. ANOVA P=0.0003. Tukey´s multiple comparisons (**) P=0.0045).


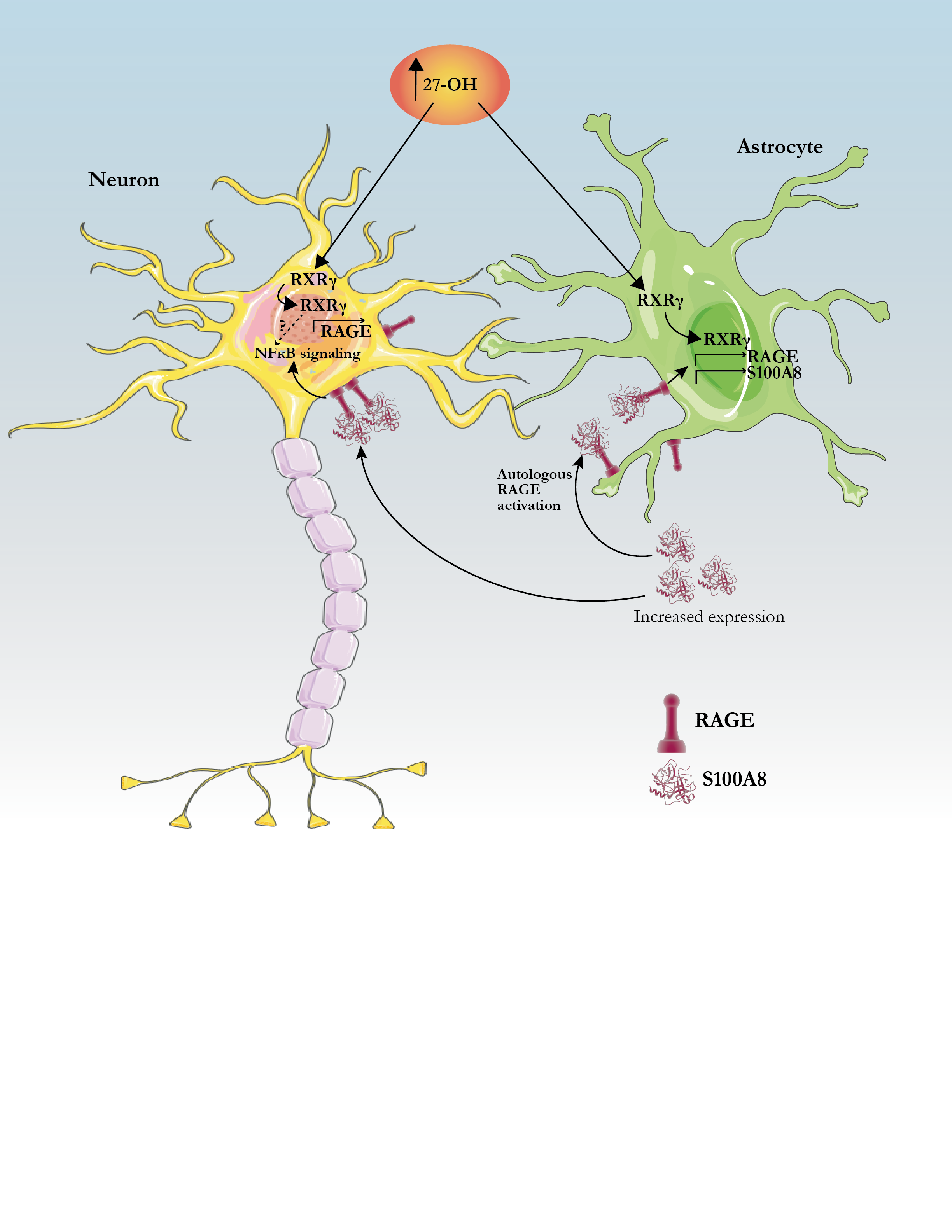


**Figure 7. Proposed mechanism of alarmin induction by 27-OH.** Elevated levels of 27-OH in the brain activate RXRγ in astrocytes, which induce expression of both RAGE and S100A8. S100A8 can then signal autologously astrocytic RAGE and further induce its expression, possibly inducing internalization of S100A8/RAGE complexes and activating astrocyte RAGE signaling for sterile inflammation. Astrocytic S100A8 activates neuronal RAGE as well, inducing alarmin signaling through NFk-B. In parallel, 27-OH can activate neuronal RXRγ inducing RAGE expression, which further sensitizes neurons to the binding of S100A8. It is unclear whether RXRγ activation by 27-OH contributes to alarmin signaling on its own (dashed arrow and question mark in the figure).


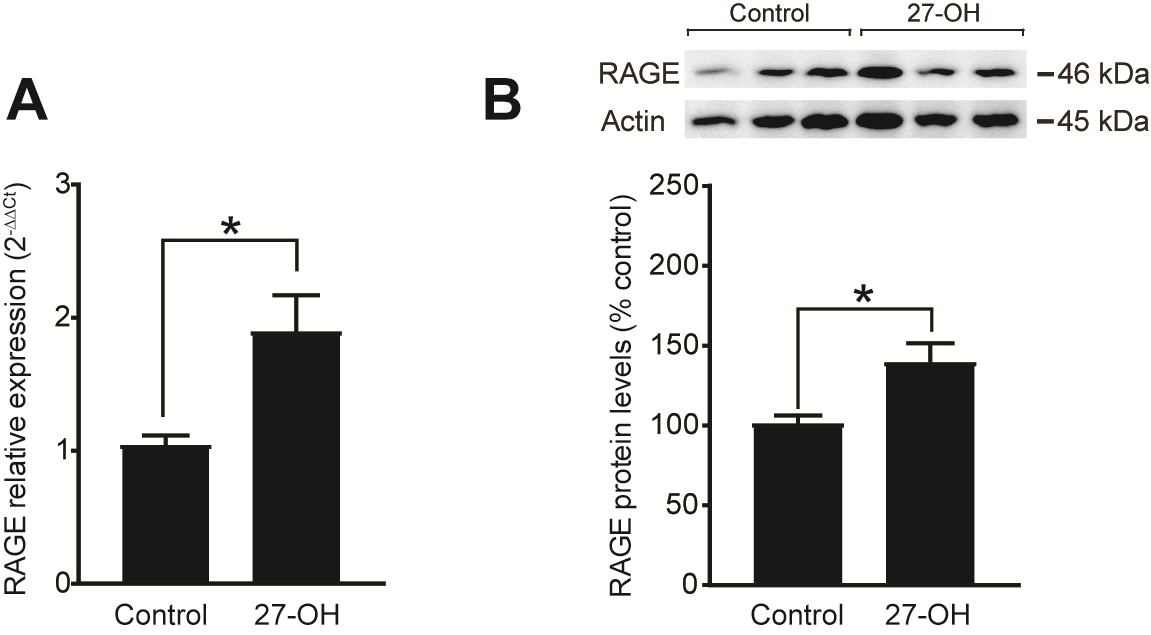


**Figure S1.** 27-OH-induced RAGE increase in human neuroblastoma cells**.** SH-SY5Y cells treated with 27-OH (10 μM, 24 h) showing both (**A)** mRNA levels of RAGE (Controlmean=1.013, SEM=0.05, n=9 wells; 27-OHmean=1.770, SEM=0.37, n=8 wells. Unpaired t test P=0.02) and (**B**) protein levels of RAGE (Controlmean=1.013, SEM=0.05, n=9 wells; 27-OHmean=1.770, SEM=0.37, n=8. Unpaired t test P=0.02).


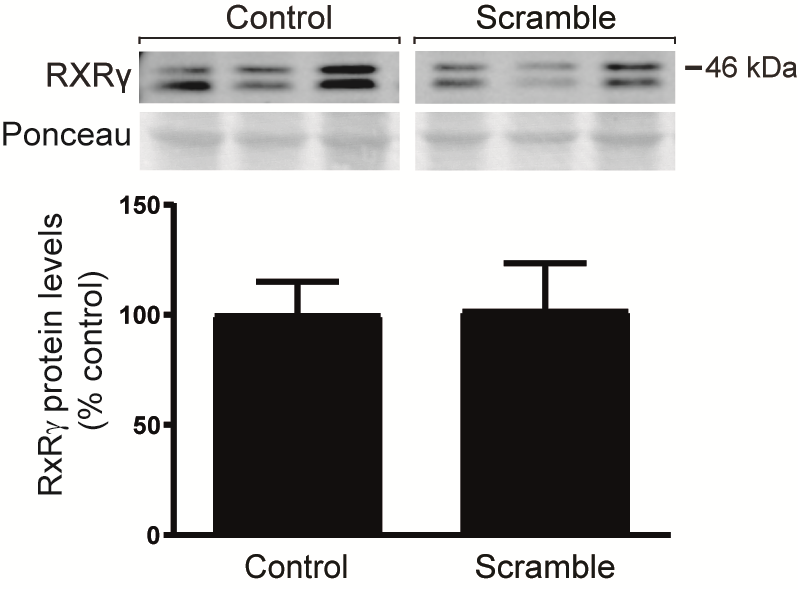


**Figure S2.** Scramble control of siRNA for RXRg shows no significant change in RXRg protein levels by activation of the siRNA system (Controlmean=100, SEM=15.01, n=3 wells; Scramblemean=101.8, SEM=21.55, n=6 wells).


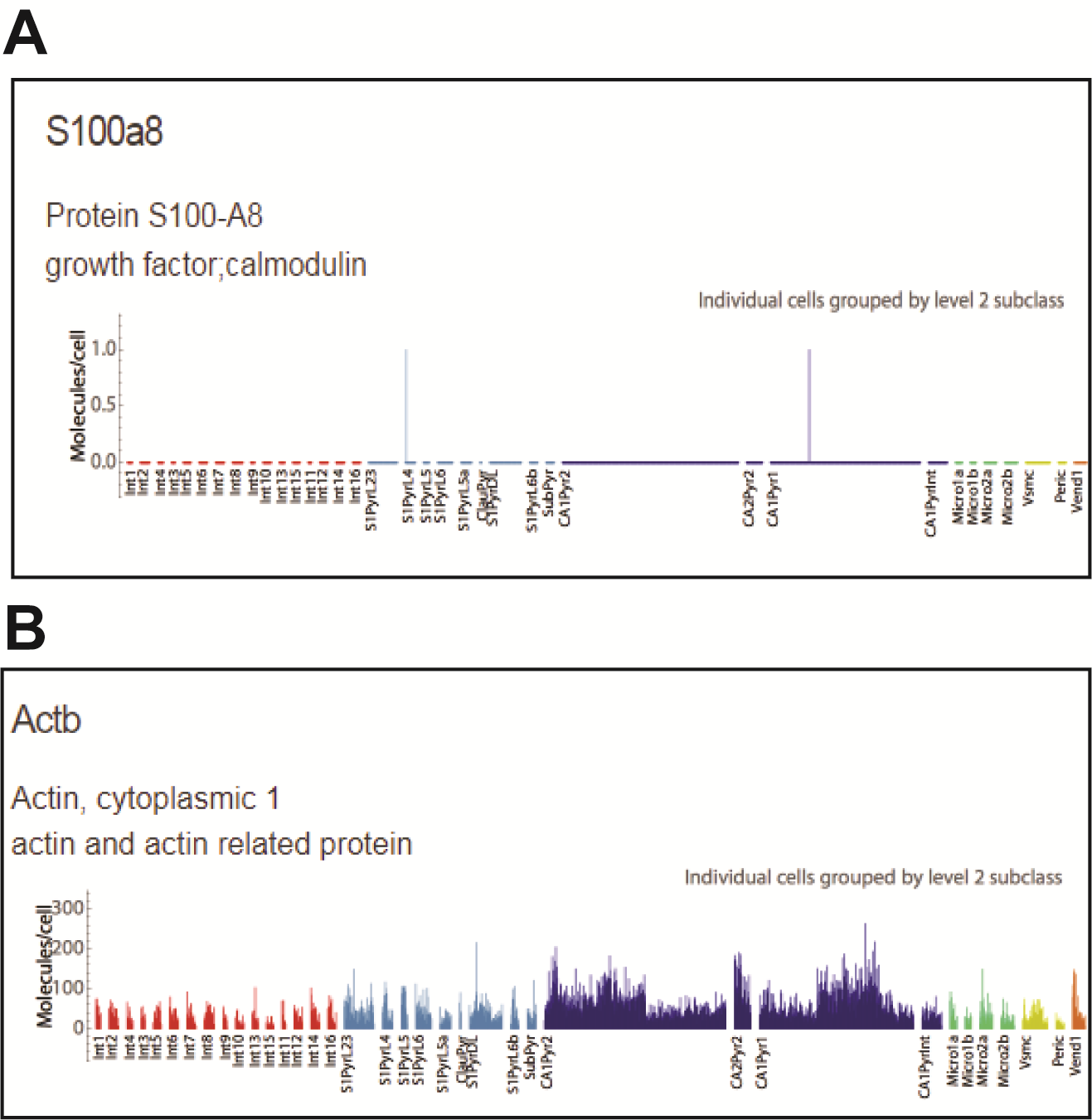


**Figure S3.** Expression of A) s100a8 and B) Actin mRNA in the mouse cortex and hippocampus by single cell sequencing. On the X axis there are single cells grouped by identity with different colors, from left to right: interneurons (red), cortical neurons (light blue), CA1 pyramidal neurons (dark blue), microglia (green), vascular smooth muscle cells (yellow), pericytes (yellow) and ependymal cells (orange). Y axis contains absolute number of RNA molecules. Note that single lines represent a cell that is expressing some copies of our gene of interest. The graph shows null expression of S100a8 in the mouse brain in a basal state compared to actin. Obtained from the database: linnarsonlab.org/cortex and reproduced with permission. For the original work, see (40)

**
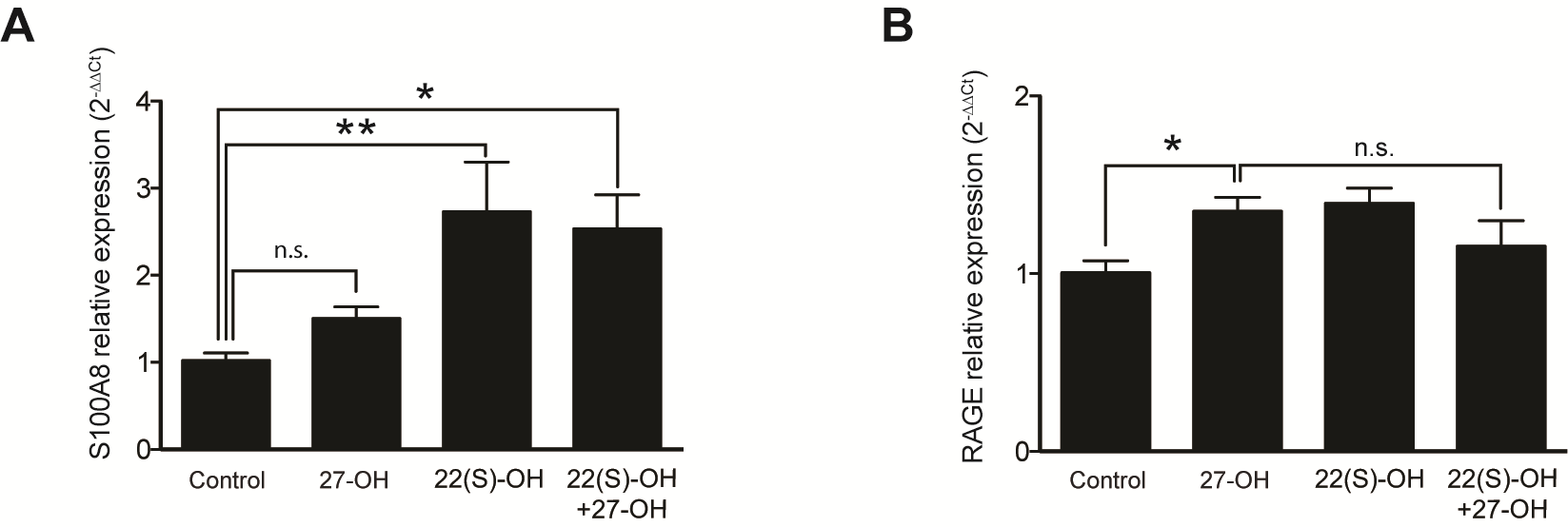
**

**Figure S4.** Blocking LXRs with 22(S)-OH do not prevent RAGE increase mediated by 27-OH. Expression of S100A8 (A) (Controlmean=1.021, SEM=0.08, n=6 wells; 27-OHmean=1.504, SEM=0.13, n=6 wells; 22(S)-OHmean=2.73, SEM=0.56, n=5 wells; 22(S)-OH+27OHmean=2.53, SEM=0.39, n=5 wells. ANOVA P=0.0038. Tukey´s multiple comparisons (*) P=0.01; (**) P=0.004; n.s. P=0.57) and RAGE (B) (Controlmean=1.006, SEM=0.06, n=4 wells; 27-OHmean=1.351, SEM=0.77, n=4 wells; 22(S)-OHmean=1.39, SEM=0.08, n=5 wells; 22(S)-OH+27OHmean=1.155, SEM=0.14, n=3 wells. ANOVA P=0.029. Tukey´s multiple comparisons (*) P=0.03, n.s. P=0.7) by real-time PCR showed that pre-incubation with 22(S)-OH (10 μM, 3 h) did not revert the effects mediated by 27-OH in glial cultures.


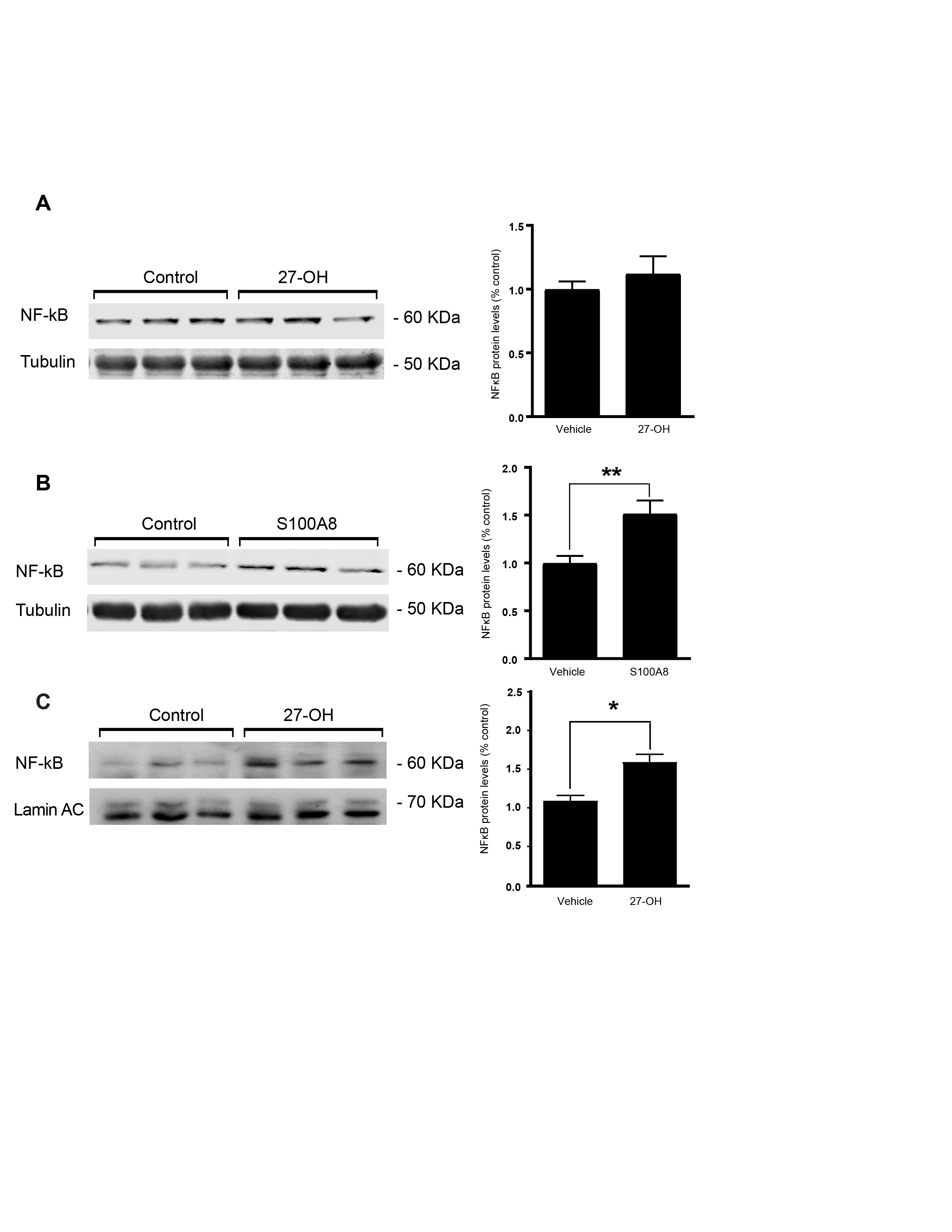


**Figure S5.** Role of NFk-B in 27-OH induction of S100A8 alarmin. (**A**) Western blots from rat primary neurons treated with 27-OH (1 μM, 24 h) showing protein levels of NFk-B (Controlmean=1.0, SEM=0.06, n=8 wells; 27-OHmean=1.122, SEM=0.13, n=8 wells. Unpaired t test P=0.42). (**B**) Western blots from rat primary cultures treated with S100A8 (12.5 ug/ml, 24h) showing protein levels of NFk-B (Controlmean=1.0, SEM=0.07, n=12 wells; 27-OHmean=1.51, SEM=0.13, n=12 wells. Unpaired t test P=0.003). **(C)** NFk-B protein levels in nuclear fractions of SH-SY5Y neuroblastoma cells treated with 27-OH (10 μM, 24 h. Controlmean=1.09, SEM=.05, n=9 wells; 27-OHmean=1.45 SEM=.09, n=9 wells. Unpaired t test P=0.0049).
